# Structural identifiability of biomolecular controller motifs with and without flow measurements as model output

**DOI:** 10.1371/journal.pcbi.1011398

**Published:** 2023-08-28

**Authors:** Eivind S. Haus, Tormod Drengstig, Kristian Thorsen

**Affiliations:** Department of Electrical Engineering and Computer Science, University of Stavanger, Stavanger, Norway; Pázmány Péter Catholic University: Pazmany Peter Katolikus Egyetem, HUNGARY

## Abstract

Controller motifs are simple biomolecular reaction networks with negative feedback. They can explain how regulatory function is achieved and are often used as building blocks in mathematical models of biological systems. In this paper we perform an extensive investigation into *structural identifiability* of controller motifs, specifically the so–called *basic* and *antithetic* controller motifs. Structural identifiability analysis is a useful tool in the creation and evaluation of mathematical models: it can be used to ensure that model parameters can be determined uniquely and to examine which measurements are necessary for this purpose. This is especially useful for biological models where parameter estimation can be difficult due to limited availability of measureable outputs. Our aim with this work is to investigate how structural identifiability is affected by controller motif complexity and choice of measurements. To increase the number of potential outputs we propose two methods for including flow measurements and show how this affects structural identifiability in combination with, or in the absence of, concentration measurements. In our investigation, we analyze 128 different controller motif structures using a combination of flow and/or concentration measurements, giving a total of 3648 instances. Among all instances, 34% of the measurement combinations provided structural identifiability. Our main findings for the controller motifs include: i) a single measurement is insufficient for structural identifiability, ii) measurements related to different chemical species are necessary for structural identifiability. Applying these findings result in a reduced subset of 1568 instances, where 80% are structurally identifiable, and more complex/interconnected motifs appear easier to structurally identify. The model structures we have investigated are commonly used in models of biological systems, and our results demonstrate how different model structures and measurement combinations affect structural identifiability of controller motifs.

## Author summary

Creating a mathematical model of a biological system can be a powerful way to gain insight into the behavior of the system. However, the accuracy and quality of model predictions depend heavily on model parameters. Compared to traditional human–engineered systems, such as mechanical or electrical systems, the availability of measurable outputs is severely limited for many biological systems. Furthermore, parameter estimation for biological models is often both challenging and associated with high cost. In this context, structural identifiability analysis is a helpful tool for finding the smallest or easiest to perform set of measurements that is sufficient to uniquely determine the model parameters. In this paper, we investigate a group of biological models called controller motifs, and we examine how varying model complexity and choice of measurements affect structural identifiability. We propose two alternative ways to include flow measurements as model output and show that structural identifiability can be achieved using a combination of concentration and/or flow measurements. Controller motifs can be used in a wide range of biological models, and our results can contribute to create structurally identifiable models.

## Introduction

In systems biology, mathematical models are used to gain insight into the behavior and function of biological systems [1–3]. The quality of model predictions is heavily dependent on both model structure and parameter values [4, 5]. Parameter values are typically estimated from experiments, either directly or from results published in literature. It is, however, well known that available measurements from biological processes often are severely limited and associated with high cost [6, 7], which make experimental design optimization important [8, 9]. Furthermore, given a set of available measurements, it is in general not straight forward to determine the smallest set of measurements that is sufficient to uniquely estimate a model’s parameters. In this context, the concept of *structural identifiability* [10–15] is a helpful tool for determining this smallest set of measurements [16, 17]. The core of the concept states that [10] “*If a model is structurally identifiable, it is theoretically possible to uniquely determine the values of its parameters by observing its outputs.*” For a model as a whole to be structurally identifiable, all parameters must be identifiable. As a consequence, it is impossible to uniquely determine at least one of the parameters of a *structurally unidentifiable* model, which may lead to erroneous predictions and a less useful model overall. In a structurally unidentifiable model a subset of the parameters is typically unidentifiable, which implies that these parameters are correlated.

The term *structural* signifies that structural identifiability is completely determined by the structure of the model, including the chosen set of measurements. A prerequisite for structural identifiability is that input perturbations provide sufficiently informative outputs/measurements [12, 18, 19]. It should also be noted that structural identifiability is a theoretical concept, and that *practical identifiability* limitations may still occur [5, 20–22]. This is however outside the scope of this paper.

One approach for creating a mathematical model is to include as many details and phenomena as possible, as this is expected to increase the model’s ability to reflect the modeled system’s behavior [23]. However, as more details and phenomena are included, the complexity of the model increases, either in the form of *i*) model size (more states, reactions, and/or parameters) or in the form of *ii*) complexity of reaction kinetic expressions. It may be more difficult to estimate parameter values of a more complex model. Thus, there will often be a trade–off between model complexity and the ability to estimate model parameters from a given set of measurements [24]. In this paper we investigate how these two dimensions of model complexity, i.e., model size and reaction kinetic expressions, affect structural identifiability.

*Controller motifs* are relatively simple biomolecular reaction networks that contain negative feedback [25–34], which make them useful as building blocks in larger regulatory networks [35–39]. Controller motifs are used to explain how regulation is achieved in biological systems, and they have recently been used in a variety of experimental implementations of control systems in genetically engineered cells [40–42]. They have been used in synthetic genetic circuits in both bacteria [43, 44] and mammalian cells [45, 46]. Different properties of controller motifs have been studied extensively, but to our knowledge and supported by an extensive literature search (see S1 Text), structural identifiability of controller motifs has so far not been investigated. Motivated by the fact that both dynamic and static performance of controller motifs are heavily affected by parameter values [26, 47–49], we investigate in this paper the structural identifiability of the so–called *basic controller motifs* [26] and the *antithetic controller motifs* [28, 50].

The available measurements from the investigated controller motifs include first and foremost the concentrations of the modeled species. Taking into consideration that wet lab experiments may involve measurements of flow instead of, or in addition to, concentration [51–55], we also want to include flow measurements as model output candidates. For this purpose, we propose two different modeling approaches for incorporating flow measurements into a model. We use these approaches to investigate structural identifiability of the controller motifs for all possible combinations of concentration and flow measurements with one or two measurements as model output.

## Models and methods

### Models

#### Basic controller motifs

**Fig 1:**
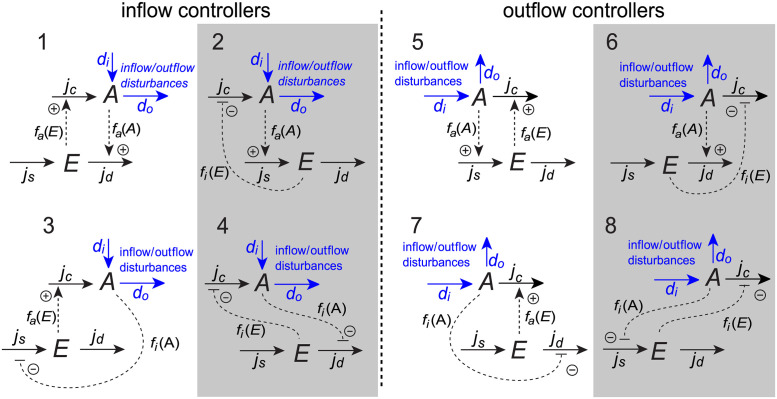
Basic controller motifs. The 8 basic controller motifs characterized as either inflow or outflow controllers. White and gray backgrounds mark activating or inhibiting motifs, respectively (see main text for definitions).

The set of basic controller motifs consists of 8 different two–component molecular reaction networks where the controlled species *A* and the controller species *E* are interconnected to achieve negative feedback and regulatory function [26], see Fig 1. The controller motifs are categorized as either *inflow* or *outflow* controllers, depending on whether the controller species *E* is compensating for disturbances by adding or removing *A*, respectively.

The main function of the compensatory flow *j*_*c*_ is to maintain a regulated level of *A* by compensating for the disturbances *d*_*i*_ and *d*_*o*_. The controller motifs are characterized as either *activating* or *inhibiting*, depending on whether *j*_*c*_ is activated or inhibited by *E*. Similarly, the signaling from *A* to *E* is either by activation or inhibition through either the synthesis flow *j*_*s*_ or the degradation flow *j*_*d*_. The homeostatic setpoint for the level of *A* depends on the rate constants of these two flow expressions [35].

The general state equations for the basic controller motifs are given as:

$$\begin{array}{c} \frac{dA(t)}{dt}=d_{i}\left(t\right)-d_{o}\left(t\right)\pm j_{c}\left(t\right) \end{array}$$ (1)

$$\begin{array}{c} \frac{dE(t)}{dt}=j_{s}\left(t\right)-j_{d}\left(t\right), \end{array}$$ (2)

where *A* and *E* are the state variables, and *d*_*i*_, *d*_*o*_, *j*_*c*_, *j*_*s*_, and *j*_*d*_ are the different flow expressions. In a practical setting the state variables *A* and *E* are usually concentrations with a typical unit of M (mol/L). Thus, the unit of the flows is rate of concentration change, i. e., M/s. Under the assumption that the reactions take place in a constant volume, which is a common assumption in the treatment of controller motifs [56], concentration flow rate is equivalent to mass flow rate. The treatment of the controller motifs in this paper is, however, purely symbolic and does not consider the choice of units.

In order to investigate how the two aforementioned dimensions of model complexity affect structural identifiability, we categorize the models for each controller motif into levels of complexity based on the following list:

1. **Disturbances.** The expressions for the inflow and outflow disturbances are given as:
  $$\begin{array}{c} \;d_{i}\left(t\right)=k_{i} \end{array}$$ (3)
  $$\begin{array}{c} \;d_{o}\left(t\right)=k_{o}\cdot A\left(t\right), \end{array}$$ (4)
  where *k*_*i*_ and *k*_*o*_ are rate constants. The main disturbance for an inflow or outflow controller is the outflow disturbance in Eq (4) or the inflow disturbance in Eq (3), respectively [35]. Model complexity, in the form of model size, is increased by including both disturbances.
2. **Activating signaling kinetics**. For controller motifs that contain activating signaling kinetics between the species, we consider two levels of reaction kinetic complexity, either first order or saturable activation kinetics. Specifically, the expression candidates for the signaling between *A* and *E* are given as:
  $$\begin{array}{c} \;f_{a}\left(A\right)=A(t) \end{array}$$ (5)
  $$\begin{array}{c} \;f_{a}\left(A\right)=\frac{A(t)}{K_{a}^{A}+A\left(t\right)}, \end{array}$$ (6)
  where $K_{a}^{A}$ is an activation constant. Similarly, the expressions for the signaling between *E* and *A* are given as:
  $$\begin{array}{c} \;f_{a}\left(E\right)=E(t) \end{array}$$ (7)
  $$\begin{array}{c} \;f_{a}\left(E\right)=\frac{E(t)}{K_{a}^{E}+E\left(t\right)}, \end{array}$$ (8)
  where $K_{a}^{E}$ is an activation constant.
3. **Kinetics in the degradation of *E*.** The candidate expressions for the degradation flow of *E* are either zero order, first order, or saturable Michaelis–Menten kinetics with respect to *E* given as:
  $$\begin{array}{c} \;j_{d}\left(t\right)=k_{d}\cdot f_{a/i}\left(A\right) \end{array}$$ (9)
  $$\begin{array}{c} \;j_{d}\left(t\right)=k_{d}\cdot f_{a/i}\left(A\right)\cdot E\left(t\right) \end{array}$$ (10)
  $$\begin{array}{c} \;j_{d}\left(t\right)=k_{d}\cdot f_{a/i}\left(A\right)\cdot \frac{E(t)}{K_{M}^{E}+E\left(t\right)}, \end{array}$$ (11)
  where *k*_*d*_ is a rate constant, $K_{M}^{E}$ is a Michaelis–Menten constant, and *f*_*a*/*i*_(*A*) is either one of the activation expressions in Eqs (5) or (6), or the inhibition expression in Eq (12) shown below.

**Fig 2:**
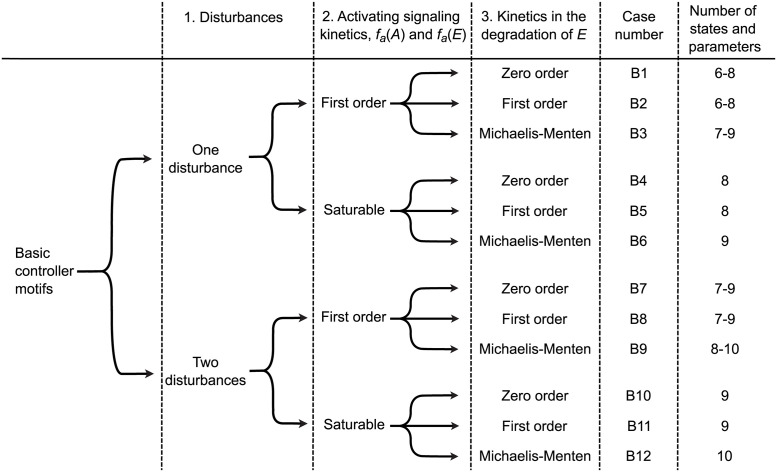
Investigated cases for the basic motifs. Organization of the investigated cases for the basic controller motifs, taking into account the number of disturbances, the different expression candidates for the activation kinetics *f*_*a*_(*A*) and *f*_*a*_(*E*), and the expression candidates for the degradation kinetics of the controller species *E*. The combinations of these model complexities result in 12 cases named B1–B12, where the total number of states and parameters varies between 6 and 10.

Thus, the levels of model complexity can be organized as shown in Fig 2, where we have 12 distinct cases labeled B1–B12. This gives a total of 96 different models for the basic controller motifs.

We use only one expression candidate for the inhibiting signaling kinetics *f*_*i*_(*E*) and *f*_*i*_(*A*), i.e.,:

$$\begin{array}{c} f_{i}\left(A\right)=\frac{K_{i}^{A}}{K_{i}^{A}+A\left(t\right)} \end{array}$$ (12)

$$\begin{array}{c} f_{i}\left(E\right)=\frac{K_{i}^{E}}{K_{i}^{E}+E\left(t\right)}, \end{array}$$ (13)

where $K_{i}^{A}$ and $K_{i}^{E}$ are inhibition constants. Thus, inhibition kinetics do not introduce different levels of complexity. Moreover, for the outflow controller motifs, the compensatory flow’s dependence on *A* is expressed as first order:

$$\begin{array}{c} j_{c}\left(t\right)=k_{c}\cdot f_{a/i}\left(E\right)\cdot A\left(t\right), \end{array}$$ (14)

where *k*_*c*_ is a rate constant and *f*_*a*/*i*_(*E*) denotes either activation or inhibition from *E*.

See supplementary S2 Text for a complete overview of system equations for all basic motifs and cases.

#### Antithetic controller motifs

**Fig 3:**
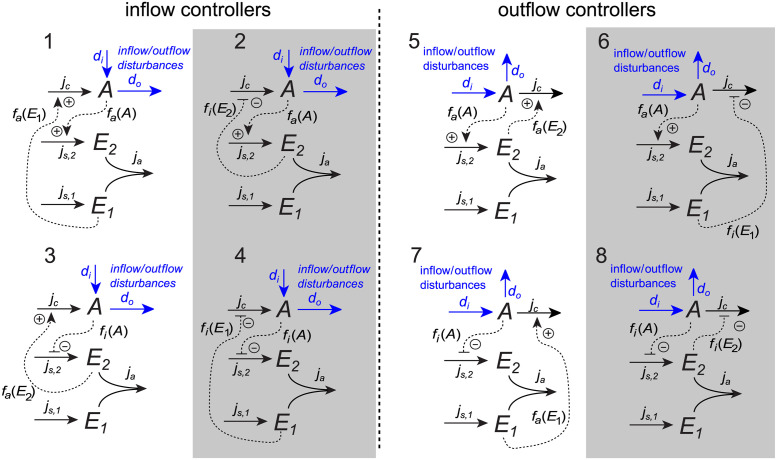
Antithetic controller motifs. The 8 antithetic controller motifs characterized as either inflow or outflow controllers. White and gray backgrounds mark activating or inhibiting motifs, respectively (see main text for definitions).

The antithetic controller motifs provide negative feedback and robust perfect adaption through integral control [28]. The first antithetic controller motif was introduced using a stochastic framework, and its characteristics has been thoroughly investigated [38, 39, 57]. In this paper we will consider the deterministic version of the antithetic controller motif, and as shown in [50] there is a set of 8 antithetic controller motifs, see Fig 3.

The antithetic controller motifs consist of two controller species, here called *E*_1_ and *E*_2_, and a controlled species *A*, resulting in a three–component molecular reaction network. The general state equations for the antithetic controller motifs are given as:

$$\begin{array}{c} \frac{dA(t)}{dt}=d_{i}\left(t\right)-d_{o}\left(t\right)\pm j_{c}\left(t\right) \end{array}$$ (15)

$$\begin{array}{c} \frac{dE_{1}\left(t\right)}{dt}=j_{s,1}\left(t\right)-j_{a}\left(t\right) \end{array}$$ (16)

$$\begin{array}{c} \frac{dE_{2}\left(t\right)}{dt}=j_{s,2}\left(t\right)-j_{a}\left(t\right). \end{array}$$ (17)

Similarly to the basic controller motifs, the flow *j*_*c*_ compensates for the disturbances *d*_*i*_ and *d*_*o*_, expressed as Eqs (3) and (4), in order to maintain a regulated level of *A*.

For the antithetic controller motifs we will, as for the basic motifs, investigate how model complexity affects structural identifiability. Thus, we consider motifs with one or two disturbances and different activation kinetics in *f*_*a*_(*A*) and *f*_*a*_(*E*_1_)/*f*_*a*_(*E*_2_), using similar expressions as in Eqs (5)–(8).

Contrary to the basic controller motifs, there is for the antithetic motifs only one expression candidate for the degradation kinetics of the controller species, *E*_1_ and *E*_2_, because both are degraded through a comparison reaction called the *annihilation* flow *j*_*a*_, given as:

$$\begin{array}{c} j_{a}\left(t\right)=k_{a}\cdot E_{1}\left(t\right)\cdot E_{2}\left(t\right). \end{array}$$ (18)

**Fig 4:**
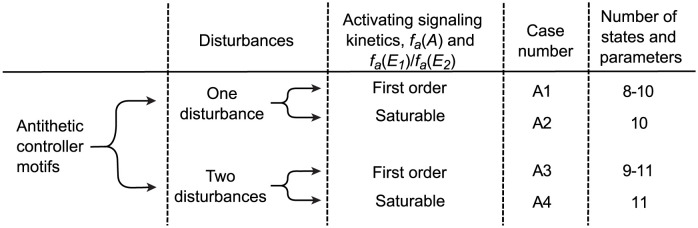
Investigated cases for the antithetic motifs. Organization of the investigated cases for the antithetic controller motifs, taking into account the number of disturbances and the different expressions for the activation kinetics *f*_*a*_(*A*) and *f*_*a*_(*E*_1_)/*f*_*a*_(*E*_2_). The combinations of these model complexities result in 4 cases named A1–A4, where the total number of states and parameters varies between 8 and 11.

Thus, the combinations of candidate expressions give 4 distinct cases as shown in Fig 4, with a total of 32 different models for the antithetic controller motifs.

See supplementary S2 Text for a complete overview of system equations for all antithetic motifs and cases.

### Methods

There exist a variety of methods to analyze structural identifiability [58–65], and each method has advantages and disadvantages with respect to ease of implementation, presentation, analysis, and computational cost [66]. To get precise a priori results we primarily want to use a symbolic method [66]. Beyond that, our main method selection criteria are simple implementation, presentation and analysis of the results due to the large number of models analyzed. Therefore, we have chosen the method that investigates structural identifiability as augmented observability, and a brief description is presented in the following.

#### Structural identifiability as augmented observability

Consider a general nonlinear state space model:

$$\begin{array}{c} \dot{x}\left(t\right)=f(x(t),u(t),p)\\ y(t)=g(x(t),p)\\ x_{0}=x(t_{0},p), \end{array}$$ (19)

where $x\left(t\right)\in R^{n}$ is the state vector, $u\left(t\right)\in R^{r}$ is the input vector, $y\left(t\right)\in R^{m}$ is the output vector, and $p\in R^{q}$ is the system parameter vector. Moreover, *x*_0_ is the vector of initial values, *f* is the vector of system equations, and finally, *g* is the vector of output functions. In our investigation we consider only autonomous systems without inputs. Therefore, the dependence on the input vector *u* will be omitted. If *f* and *g* are both infinitely differentiable analytic functions, we can obtain information about the states *x* through differentiation of the output *y*. The derivatives of *y* are found by taking the Lie derivative of the output function *g* along the system equations *f* as given by Eq (20):

$$\begin{array}{c} {£}_{f}g\left(x\right)=\frac{\partial g(x)}{\partial x}\cdot f\left(x\right). \end{array}$$ (20)

Successive Lie derivatives of order *k* are found recursively from:

$$\begin{array}{ccc} {£}_{f}^{2}g\left(x\right) & = & \frac{\partial {£}_{f}g\left(x\right)}{\partial x}\cdot f\left(x\right)\\ & \vdots \\ {£}_{f}^{k}g\left(x\right) & = & \frac{\partial {£}_{f}^{k-1}g\left(x\right)}{\partial x}\cdot f\left(x\right). \end{array}$$ (21)

In order to also obtain information about the system parameters, they are considered as additional states without dynamics, and they are included in an augmented state vector $\tilde{x}=\left[x,p\right]\in R^{n+q}$. By stacking the successive Lie derivatives with respect to the augmented state vector $\tilde{x}$ on top of each other, we obtain the so–called *generalized observability–identifiability matrix* $O_{I}\left(\tilde{x}\right)$ as [63]:

$$\begin{array}{c} O_{I}\left(\tilde{x}\right)=[\begin{array}{c} \frac{\partial }{\partial \tilde{x}}\left(g\left(\tilde{x}\right)\right)\\ \frac{\partial }{\partial \tilde{x}}\left({£}_{f}g\left(\tilde{x}\right)\right)\\ \frac{\partial }{\partial \tilde{x}}\left({£}_{f}^{2}g\left(\tilde{x}\right)\right)\\ \vdots \\ \frac{\partial }{\partial \tilde{x}}\left({£}_{f}^{\left(n+q-1\right)}g\left(\tilde{x}\right)\right) \end{array}]. \end{array}$$ (22)

A generalized condition for observability and identifiability is defined as follows [63]:*“If the system given by* Eq (19) *satisfies rank* ($O_{I}\left(\tilde{x}\right))=n+q$, *then the system is (locally) observable and identifiable in a neighborhood* $N({\tilde{x}}_{0})$ *of* ${\tilde{x}}_{0}$.” The rank condition only provides results for local structural identifiability (and observability), but in many practical applications this also implies global structural identifiability [14].

It should be mentioned as a sidenote that this method of testing structural identifiability may sometimes produce results that do not hold for some initial conditions [67–69], e.g., if some state values are unreachable from specific initial conditions. An example of such is the autocatalytic controller motif (not considered here) which has an absorbing state if the controller species *E* is zero [70, 71]. Hence, *E* will permanently remain at 0 and states outside the *E* = 0 subspace are thus unreachable. Methods to detect problematic initial conditions by substituting numerical values for the symbolic states have been proposed [60, 68]. However, we do not pursue this further in this paper.

The computational cost of evaluating the rank of $O_{I}\left(\tilde{x}\right)$ increases with the number of states and parameters, as higher order Lie derivatives must be computed, and thus, the dimensions and complexity of $O_{I}\left(\tilde{x}\right)$ increase. The symbolic rank operation on the increasingly large and complex $O_{I}\left(\tilde{x}\right)$ matrix accounts for most of the computational cost, both in terms of memory requirement and execution time. As shown in Eq (22), the maximum number of necessary Lie derivatives to build the $O_{I}\left(\tilde{x}\right)$ matrix is (*n*+*q*−1). However, since each successive Lie derivative can include several rows, depending on the number of measurements, fewer Lie derivatives are sometimes sufficient to establish the correct rank of $O_{I}\left(\tilde{x}\right)$, consequently reducing the computational cost. In order to determine whether $O_{I}\left(\tilde{x}\right)$ has been built with a sufficient number of Lie derivatives, one of the following three conditions must be met [63, 72]:

1.
$O_{I}\left(\tilde{x}\right)$ has full rank with the current number of Lie derivatives.
2.
$O_{I}\left(\tilde{x}\right)$ is rank deficient and rank$\left(O_{I}{\left(\tilde{x}\right)}_{k}\right)$ = rank$\left(O_{I}{\left(\tilde{x}\right)}_{k-1}\right)$ where *k* is the number of Lie derivatives. Adding more Lie derivatives beyond this point will not increase the rank of $O_{I}\left(\tilde{x}\right)$.
3.
$O_{I}\left(\tilde{x}\right)$ is rank deficient and the maximum number of Lie derivatives, (*n*+*q*−1), has been reached. Adding more Lie derivatives beyond this point will not increase the rank of $O_{I}\left(\tilde{x}\right)$.

Having described our chosen method of analyzing structural identifiability, we continue by presenting the two alternative modeling approaches for including flow measurements as model output.

#### Expanding a model with flows added as states (method 1)

For models of biological processes, the output signals *y* are typically some internal states *x*, which usually are concentrations of chemical species such as proteins or metabolites. Examples of such models include the JAK/STAT signaling pathway and the NF–*κ*B pathway [73, 74], which are commonly used as benchmark models for structural identifiability [11, 13, 60, 66]. As flows in general are not state variables, the approach in method 1 is to expand the state vector *x* by including the flow as a state, and thus, making it available as a measurement in *y*.

Note that expanding the state vector *x* increases the total number of states and parameters to be identified. As this is expected to affect structural identifiability, we eliminate one parameter in the candidate flow expression, typically the rate constant, by substituting it with the new state variable. This compensates for an unintended increase in parameters, and in the augmented state vector $\tilde{x}$ the rate constant related to the original flow expression is substituted with the new state. Thus, the number of elements in $\tilde{x}$, and thereby the number of states and parameters to be identified, is maintained.

**Fig 5:**
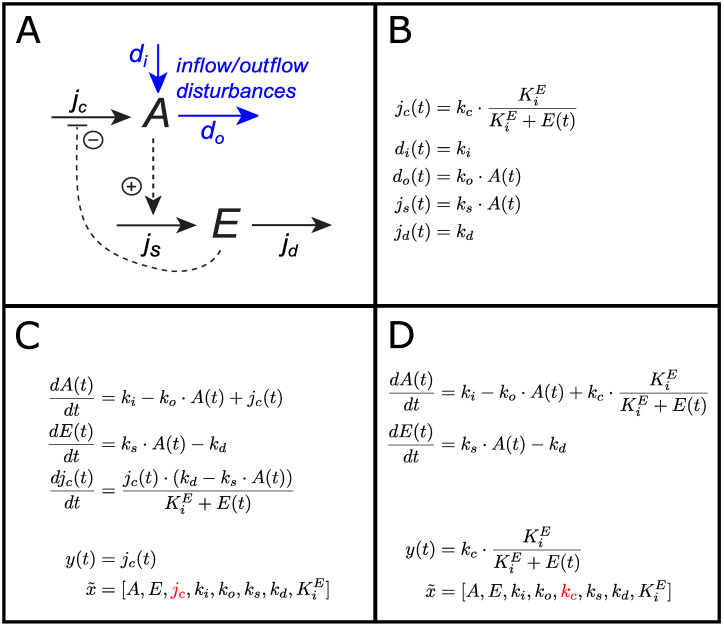
Comparison of methods 1 and 2 for adding flow measurements. Panels A and B: Schematic of basic controller motif 2 and the corresponding flow expressions for case B7. Panels C and D: Method 1 and method 2, respectively, for including flow measurements as model output. State equations, model output, and $\tilde{x}$ are shown. The total number of states and parameters to be identified (number of elements in $\tilde{x}$) is equal for both methods, though method 1 has the state, *j*_*c*_, whereas method 2 has the original system parameter, *k*_*c*_ (both marked in red).

We illustrate this method using motif 2, shown in Fig 5A, where we include the compensatory flow *j*_*c*_ as a state. The original expression for *j*_*c*_ is:

$$\begin{array}{c} j_{c}\left(t\right)=k_{c}\cdot \frac{K_{i}^{E}}{K_{i}^{E}+E\left(t\right)}, \end{array}$$ (23)

where *k*_*c*_ is a rate constant and $K_{i}^{E}$ is an inhibition constant. The derivative of *j*_*c*_ with respect to time gives the state equation for *j*_*c*_ as:

$$\begin{array}{c} \frac{dj_{c}\left(t\right)}{dt}=\frac{-k_{c}\cdot K_{i}^{E}}{{\left(K_{i}^{E}+E\left(t\right)\right)}^{2}}\cdot \frac{dE(t)}{dt}, \end{array}$$ (24)

where $\frac{dE(t)}{dt}$ is the state equation for the concentration of *E*. The expression for the parameter *k*_*c*_ can be found from Eq (23) as:

$$\begin{array}{c} k_{c}=\frac{j_{c}\left(t\right)\cdot \left(K_{i}^{E}+E\left(t\right)\right)}{K_{i}^{E}}, \end{array}$$ (25)

and together with the state equation for *E*:

$$\begin{array}{c} \frac{dE(t)}{dt}=k_{s}\cdot A\left(t\right)-k_{d}, \end{array}$$ (26)

we substitute both into Eq (24) and find the new state expression for *j*_*c*_ as:

$$\begin{array}{c} \frac{dj_{c}\left(t\right)}{dt}=\frac{j_{c}\left(t\right)\cdot \left(k_{d}-k_{s}\cdot A\left(t\right)\right)}{K_{i}^{E}+E\left(t\right)}. \end{array}$$ (27)

We note that the parameter *k*_*c*_ is no longer part of the model. However, it can be calculated from Eq (25), assuming that all the elements in the equation are identifiable/observable. The state equations for motif 2 when the compensatory flow *j*_*c*_ is added as a state is shown in Fig 5C. Note that the flow expression for *j*_*c*_, originally present in the state equation for *A*, is replaced by a single state variable. As *j*_*c*_ is a part of the state vector *x*, it can be chosen as model output *y*. The approach described here can be used to include any flow in the state vector of a model, facilititating the combined use of concentration and/or flow measurements in the measured output *y*.

#### Including flow expressions explicitly in measured output (method 2)

An alternative method of including flow measurements as outputs, is to use the flow expression directly in the expression for the measured output *y*. This results in a more complex output function, but it does not alter the augmented state vector $\tilde{x}$ as in method 1.

Continuing with motif 2 as an example, Fig 5D shows the state equations for the concentrations *A* and *E*. The expression for *y* is now the flow expression for *j*_*c*_ shown in Fig 5B. In the same way as for method 1, any combination of concentration and/or flow measurements can be included in the measured output *y*.

Supplementary S2 Text contains additional examples where flows other than *j*_*c*_ are used in the measured output *y*.

#### Our algorithm

Our algorithm is implemented to allow for easy selection of different measurement combinations as model output. The output from our algorithm is the rank of $O_{I}\left(\tilde{x}\right)$, where a rank number equal to the number of states and parameters in the model indicates structural identifiability and observability. In order to compare method 1 and 2 with respect to execution time and outcome, we programmed our algorithm to run through all the different measurement combinations for each motif and case using both methods. For each run, we stored the execution time and the rank. A summary of the algorithm is as follows:

1. Initialization: Define flow expressions *j*_*i*_(*x*(*t*), *p*) and state equations *f*(*x*(*t*), *p*) for the selected motif and case number.
  - Method 1: Adding flows as states in the model:
    – Calculate the flow state equations through differentiation of the flow expressions and perform substitution of parameters.
    – Compile a set *M* of all combinations of concentration and flow measurements to be tested. Add candidate flows as states and select the corresponding states in the output function *g*.
  - Method 2: Including flow expressions explicitly in the output function:
    – Compile a set *M* of all combinations of concentration and flow measurements to be tested. Add corresponding state variables or flow expressions explicitly into the output function *g*.
2. Computation:
  for each method
   for each measurement combination in M
    for *k* = 1→*n* + *q* − 1
     Calculate Lie derivative ${£}_{f}^{k}g\left(x\right)$
     Construct $O_{I}\left(\tilde{x}\right)$
     Calculate rank($O_{I}\left(\tilde{x}\right)$)
     if $O_{I}\left(\tilde{x}\right)$ has full rank
      break, model is identifiable
     if rank(k-1) = rank(k)
      break, model is unidentifiable
    store rank number in a table
3. If $O_{I}\left(\tilde{x}\right)$ has full rank, i.e., the rank is equal to the *n* + *q* states and parameters to be identified, the model is structurally identifiable and observable with the current set of measurements. If this condition is not met, the model is structurally unidentifiable.

## Results

For each basic controller motif we investigated the 12 cases shown in Fig 2, which results in 96 different model structures. Similarly, for each antithetic controller motif we investigated the 4 cases shown in Fig 4, which results in 32 different model structures. For each model structure we analyzed structural identifiability using either one or two measurements from the set of possible concentration measurements (*A* and *E*) and the set of possible flow measurements, i.e.,:

- *d*_*i*_, *d*_*o*_, *j*_*c*_, *j*_*s*_, and *j*_*d*_ for the basic controller motifs.
- *d*_*i*_, *d*_*o*_, *j*_*c*_, *j*_*s*,1_, *j*_*s*,2_, and *j*_*a*_ for the antithetic controller motifs.

For the basic motifs this amount to 21 or 28 measurement combinations analyzed for motifs with one or two disturbances, respectively. For the antithetic motifs this amount to 36 or 45 measurement combinations analyzed for motifs with one or two disturbances, respectively. This gives in total 3648 *instances* of model structure and output combinations. In the following we use the term *parameters* to refer to the number of both states and system parameters in $\tilde{x}$.

Before we present the results, we will present the computational setup.

### Computational setup

All code was written and executed in MATLAB using the Symbolic Toolbox. Computations were performed on a desktop computer with reasonable specifications, including an Intel Core i7–9700 processor @ 3 GHz, 32 GB of RAM and 50 GB of available virtual memory. Code was not optimized for parallell computing or utilization of multiple cores.

**Fig 6:**
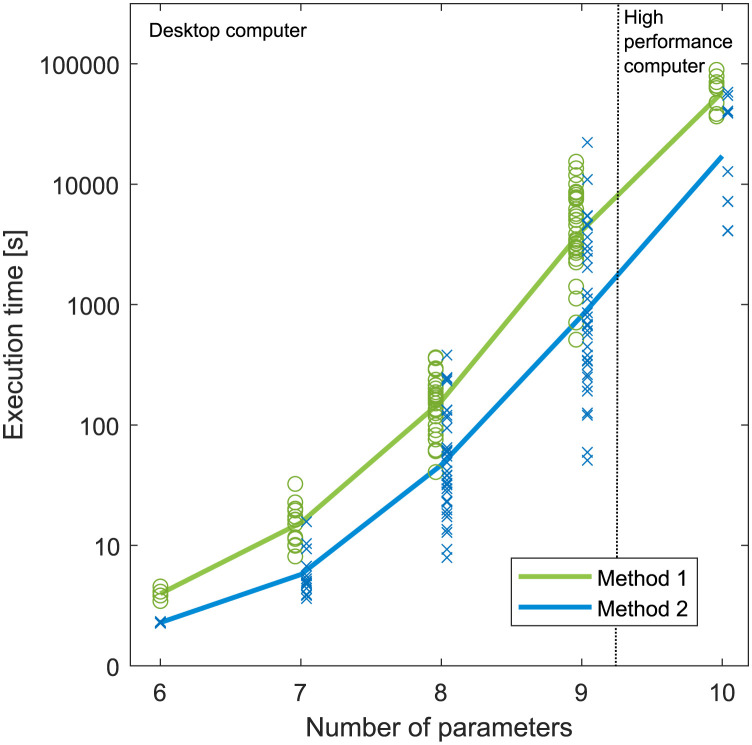
Execution times. Execution time as a function of parameters (note the use of logarithmic scale). Individual data points are marked with ∘ and ×, and the lines correspond to average values. Models with 6–9 parameters were computed on a desktop computer, whereas models with 10 parameters were computed on a high performance computer, see main text for details.

Symbolic methods for structural identifiability are known to be computationally resource demanding and time–consuming [66]. Especially memory requirements are high and increase drastically with an increase of parameters. For the basic motifs, execution times varied between a few seconds for motifs with 6 parameters and up to several hours for motifs with 9 parameters. The execution times are shown in Fig 6, which shows that the increase in execution time is close to exponential when the number of parameters increases. Method 1 shows on average longer execution times than method 2, due to increased complexity in the flow state equations from differentiation of flow expressions.

For the basic motifs with 10 parameters, the available memory on the desktop computer was insufficient, and hence, these motifs were analyzed using a high performance computer with an Intel Xeon E5–2640 v4 CPU @ 2.40 GHz and 264 GB of available memory. Motifs with 10 parameters show execution times of up to a day, a large increase from motifs with 9 parameters (computed on different platforms, not directly comparable).

For a few instances for the motifs with 10 parameters, primarily using method 1, the available memory on the high performance computer also proved insufficient for symbolic rank calculations of $O_{I}\left(\tilde{x}\right)$. For these instances, the Lie derivatives were computed symbolically, but the rank of $O_{I}\left(\tilde{x}\right)$ was evaluated numerically. To avoid accidental numerical cancellations that would reduce the rank of $O_{I}\left(\tilde{x}\right)$, we substituted the symbolic variables with uniformly distributed random numbers between 0.5 and 1.5. In addition, for each instance we executed the algorithm 10 times with different sets of random numbers. For all instances where the rank was numerically evaluated, we found that numerical calculations provided consistent results that were identical to symbolic results found with method 2.

The antithetic motifs were all analyzed using the high performance computer. Antithetic motifs with 10 parameters encountered no issues with symbolic calculations, likely because the model structures are generally simpler compared to the basic motifs with 10 parameters. However, for antithetic motifs with 11 parameters many combinations of measurements could not be analyzed symbolically due to insufficient memory. Similarly to the basic motifs, the rank calculation for these instances was performed numerically.

The full code used in the computations is available in supplementary S3 File.

### Organization of the results

**Fig 7:**
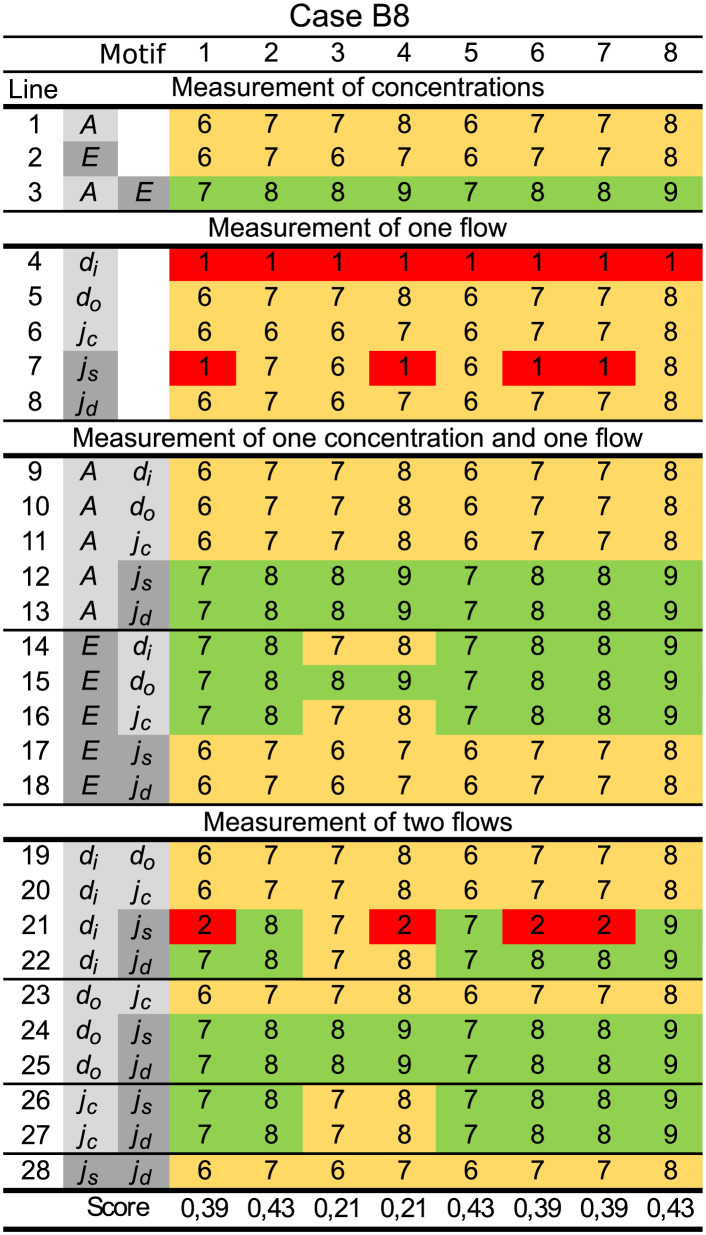
Rank of $O_{I}\left(\tilde{x}\right)$ for all motifs and measurements for case B8. The figure is organized into 4 sections, i.e., *Measurement of concentrations*, *Measurement of one flow*, *Measurement of one concentration and one flow,* and finally, *Measurement of two flows*. The first column is the line number, whereas the next two columns show the measurement combinations using grayscale colors (light gray is related to *A* and dark gray is related to *E*). The next 8 columns show the ranks of $O_{I}\left(\tilde{x}\right)$ from Eq (22) for each motif. The last row summarizes the identifiability score for each motif as the relative number of identifiable instances (green cells) in that column, see Eq (28).

**Fig 8:**
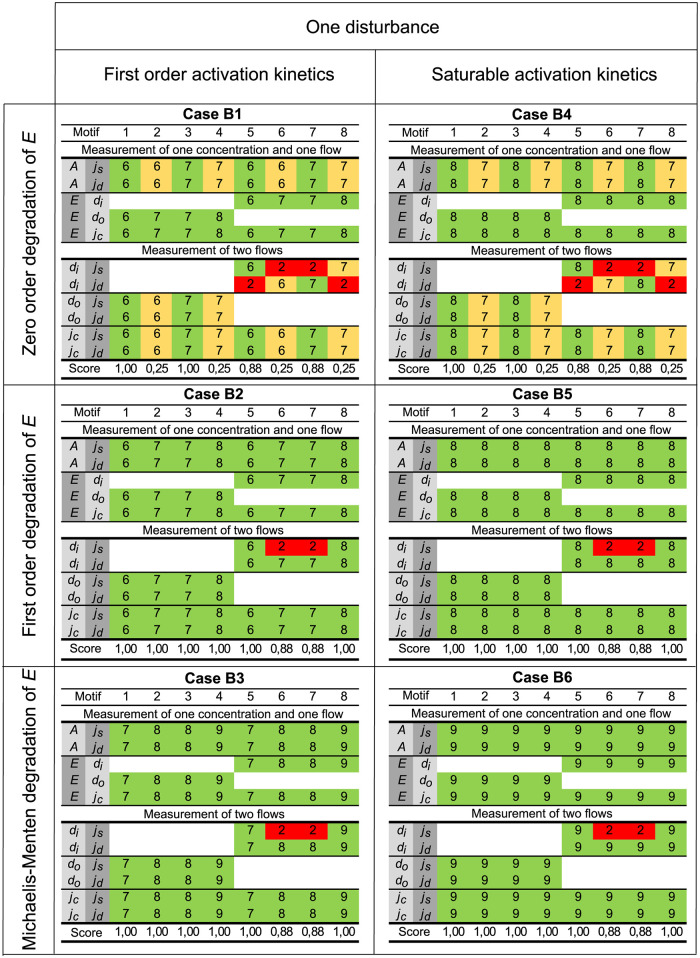
Rank of $O_{I}\left(\tilde{x}\right)$ for cases with one disturbance. The results are divided into 3 rows with 2 subpanels in each row. Each panel corresponds to a specific case number as shown in Fig 2. Results for first order and saturable activation kinetics (Eqs (5)–(8)) are found on the left and right hand side, respectively. The rows correspond to different expressions for the degradation of *E* (Eqs (9)–(11)). Within each subpanel the figure is organized as Fig 7. Only the *Case and motif dependent results* are shown.

**Fig 9:**
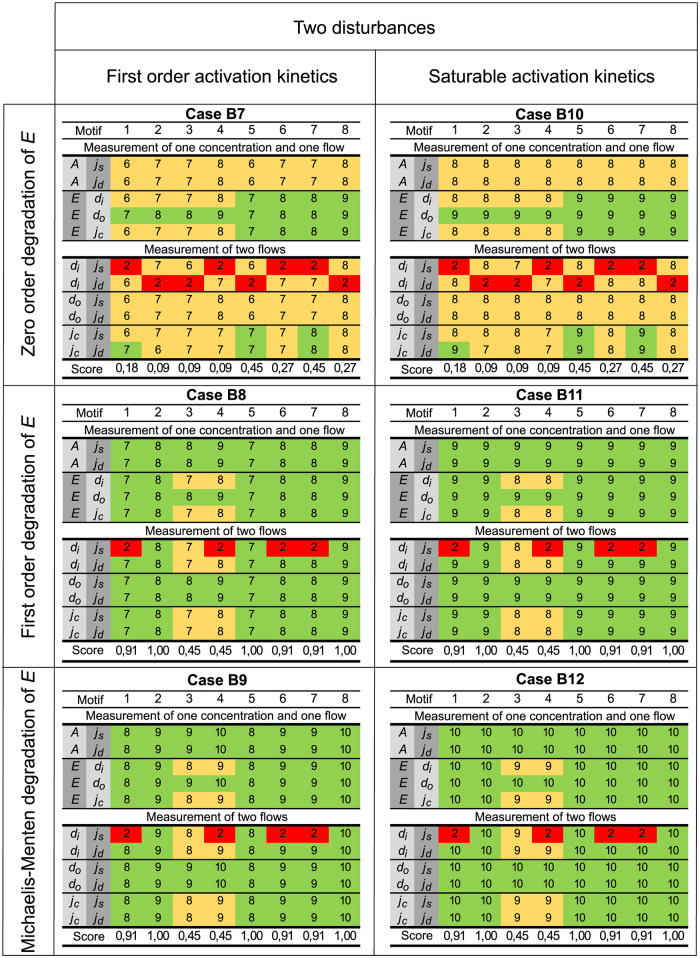
Rank of $O_{I}\left(\tilde{x}\right)$ for cases with two disturbances. The results are divided into 3 rows with 2 subpanels in each row. Each panel corresponds to a specific case number as shown in Fig 2. Results for first order and saturable activation kinetics (Eqs (5)–(8)) are found on the left and right hand side, respectively. The rows correspond to different expressions for the degradation of *E* (Eqs (9)–(11). Within each subpanel the figure is organized as Fig 7. Only the *Case and motif dependent results* are shown.

**Fig 10:**
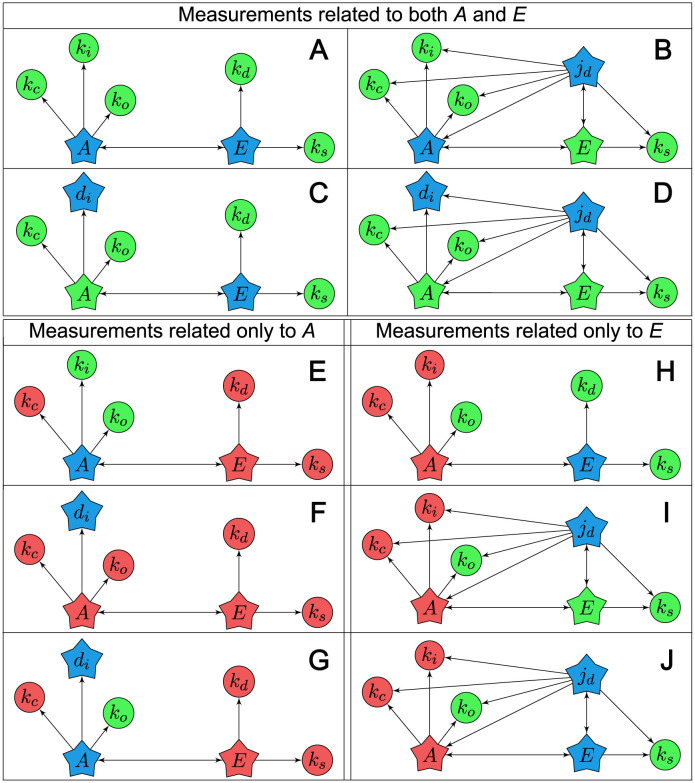
Directed graphs of selected instances from motif 1 case B8. The graphs show connections between states (stars) and system parameters (circles). For those combinations that use flow as measurement, the flow is modeled as a state according to method 1. A directed arrow from node *X* to node *Y* indicates that *Y* appears in the equation of *X*. Blue indicates measurement, green indicates an identifiable/observable parameter/state, and red indicates an unidentifiable/unobservable parameter/state. Panels A–D: Measurements related to both *A* and *E*. Panels E–G: Measurements related to *A*. Panels H–J: Measurements related to *E*.

**Fig 11:**
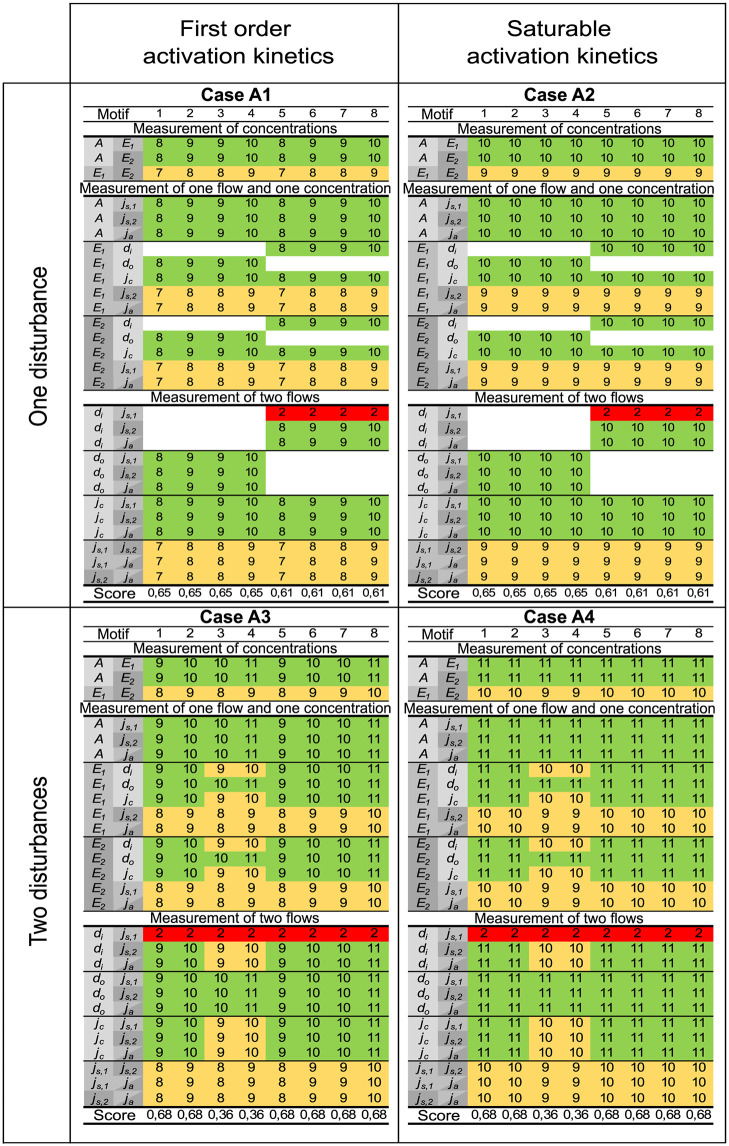
A selection of results for the antithetic motifs. The results are divided into 4 subpanels, each corresponding to a specific case number. Results for first order and saturable activation kinetics are found on the left and right hand side, respectively. Results for one and two disturbances are found in the upper and lower part, respectively. Each subpanel is organized similarly to the results for the basic motifs, see Figs 7–9. A single measurement or two measurements related to the same species, are always insufficient for structural identifiability and these results are omitted from the figure.

The results, reported as the rank of $O_{I}\left(\tilde{x}\right)$ in Eq (22), are presented in Figs 7–11 and Tables 1–3. Full rank implies that the model is structurally identifiable and observable, and the corresponding cell is colored green. If the model is structurally unidentifiable we use yellow or red. The red color is solely used when the rank is 1 or 2, which corresponds to only the chosen measurements being observable. In the following we do not differentiate between whether structural unidentifiability or unobservability is the cause of rank deficiency of $O_{I}\left(\tilde{x}\right)$.

As our aim with this study has been to investigate how structural identifiability is affected by the two dimensions of model complexity and choice of measurements, we will analyze the results with respect to the underlying motif structure. We used both methods for including flows as a measurement as shown in Fig 5, and since both methods gave identical results, we present the results as one.

To do a quantitative comparison, we define an *identifiability score* which is the relative number of identifiable instances (green cells) for each motif and case:

$$\begin{array}{c} \text{identifiability}\;\text{score}=\frac{\text{number}\;\text{of}\;\text{identifiable}\;\text{instances}}{\text{total}\;\text{number}\;\text{of}\;\text{instances}\;\text{analyzed}} \end{array}$$ (28)

### Basic controller motifs

We start by presenting the results for all possible measurement combinations for all 8 motifs for a single case. Interestingly, some of the results are general across all case platforms, and we have chosen case B8 to present these general findings, see Fig 7.

As a means to structure the results we use different grayscale colors to indicate measurements related to species *A* and *E*. From Eq (1) we note that species *A* and the flows *d*_*i*_, *d*_*o*_, and *j*_*c*_ are related, and these are colored light gray. Similarly, from Eq (2) we note that species *E* and the flows *j*_*s*_ and *j*_*d*_ are related, and these are colored dark gray.

A summary of the findings applicable for all cases, here illustrated using Fig 7, is given below. We classify the measurement combinations as either *Always insufficient*, *Always sufficient*, or *Case and motif dependent* in order to obtain structural identifiability.

- *Always insufficient*:
  – one concentration measurement (lines 1–2).
  – one flow measurement (lines 4–8).
  – one flow and one concentration measurement related to the same species (lines 9–11 and 17–18).
  – two flow measurements related to the same species (lines 19–20, 23, and 28).
- *Always sufficient*:
  – measurement of both concentrations (line 3).
- *Case and motif dependent*:
  – one flow measurement related to one species and one concentration measurement of the other species (lines 12–16).
  – two flow measurements, related to separate species (lines 21–22 and 24–27).

Based on these findings we present in Figs 8 and 9 the *Case and motif dependent* results using one disturbance (cases B1–B6, Fig 8) and two disturbances (cases B7–B12, Fig 9). Results that are *Always insufficient* or *Always sufficient* are omitted. A qualitative, visual based inspection reveals three immediate findings:

- Two disturbances result in relatively fewer green cells compared to one disturbance, and this result is rather intuitive (Fig 8 versus Fig 9).
- Models with simpler kinetic expressions show more yellow cells than models with more complex expressions (B1/B4 versus B2/B3/B5/B6 in Fig 8, and B7/B10 versus B8/B9/ B11/B12 in Fig 9). This is a more intriguing result, but not entirely unexpected [75–77].
- Measuring flows is for many motifs and cases equivalent to measuring concentrations.

Looking at all the results, *d*_*o*_ is the single best flow measurement, as measurement of *d*_*o*_ together with *E* proved sufficient for structural identifiability for all motifs and cases. This result indicates that if flow measurements are used, flows connected to species concentrations through only a constant parameter, e.g., *d*_*o*_(*t*) = *k*_*o*_⋅*A*(*t*), are best for structural identifiability.

We will in the next two sections compare the results from Figs 8 and 9 in more detail. As an overview we have calculated some aggregated measures of the identifiability score in Eq (28) for each case and motif, shown in Tables 1 and 2, respectively.

**Table 1:** Average identifiability score for each case.

	One disturbance *d*_*i*_/*d*_*o*_		Two disturbances *d*_*i*_ and *d*_*o*_	
	First order activation kinetics	Saturable activation kinetics, $K_{a}^{A}$/$K_{a}^{E}$	First order activation kinetics	Saturable activation kinetics, $K_{a}^{A}$/$K_{a}^{E}$
Zero order degradation of *E*, *k*_*d*_	Case B1 (6–8) **0.59**	Case B4 (8) **0.59**	Case B7 (7–9) **0.24**	Case B10 (9) **0.24**
First order degradation of *E*, *k*_*d*_	Case B2 (6–8) **0.97**	Case B5 (8) **0.97**	Case B8 (7–9) **0.83**	Case B11 (9) **0.83**
Michaelis–Menten degradation of *E*, *k*_*d*_ and $K_{M}^{E}$	Case B3 (7–9) **0.97**	Case B6 (9) **0.97**	Case B9 (8–10) **0.83**	Case B12 (10) **0.83**

Average scores from Figs 8 and 9 for each case. Number of parameters are given in parentheses. Complexity in terms of model size increases from the left– to the right–hand side (more disturbances), while complexity in terms of kinetic expressions increases both in the left/right and in the top/down directions within the subtables for one and two disturbances. For explanation of the colored cells, see main text.

**Table 2:** Average identifiability scores for each motif.

	Inflow controllers				Outflow controllers			
	Motif 1	Motif 2	Motif 3	Motif 4	Motif 5	Motif 6	Motif 7	Motif 8
	**One disturbance**							
Average score case B1–B6	1 (6–9)	0.75 (7–9)	1 (7–9)	0.75 (8–9)	0.96 (6–9)	0.75 (7–9)	0.88 (7–9)	0.75 (8–9)
Average for the controller type	0.88				0.84			
Zero order degradation of *E*	1	0.25	1	0.25	0.88	0.25	0.88	0.25
First order or Michaelis–Menten degradation of *E*	1	1	1	1	1	0.88	0.88	1
	**Two disturbances**							
Average score case B7–B12	0.66 (7–10)	0.70 (8–10)	0.33 (8–10)	0.33 (9–10)	0.82 (7–10)	0.70 (8–10)	0.76 (8–10)	0.76 (9–10)
Average for the controller type	0.51				0.76			
Zero order degradation of *E*	0.18	0.09	0.09	0.09	0.45	0.27	0.45	0.27
First order or Michaelis–Menten degradation of *E*	0.91	1	0.45	0.45	1	0.91	0.91	1

Average scores from Figs 8 and 9. Number of parameters are given in parantheses. The first row shows the average score for each motif for one disturbance (case B1–B6). The second row shows the average of the 4 inflow or 4 outflow controllers from the first row. The third and fourth rows show the average score for the models where the expression for the degradation of *E* follows either zero order or first order / Michaelis–Menten kinetics, respectively. The cases with two disturbances (case B7–B12) follows below in rows 5–8, and the table organization is identical. Gray–colored cells represent inhibiting motifs, whereas non–colored cells represent activating motifs.

#### Comparison of cases

Looking at the aggregated results in Table 1, together with the details in Figs 8 and 9, we can summarize the findings as follows:

- There is an overall reduction in identifiability score when one more disturbance, *d*_*i*_ or *d*_*o*_, is added to the model (illustrated by the drop in score from the blue to the orange cells in Table 1). This is a rather intuitive result as an increased number of parameters, due to more flows in the model, is more difficult to identify given the same measurements.
- A comparison of the first and second rows in Table 1 show that an increase in model complexity going from zero order to first order degradation of *E*, i.e., from Eqs (9) to (10), leads to a drastic increase in identifiability score. We note that the increase in model complexity is due to the state variable *E* appearing in its own state equation, and we use the term *self–coupling* for this dependency. Thus, increased self–coupling appears beneficial for structural identifiability.
  The observed increase in identifiability score can also be explained from the viewpoint of symmetries. The existence of Lie symmetries is equivalent to structural unidentifiability [78–80]. The zero order term in cases B1, B4, B7, and B10 might lead to simple symmetries that can be broken by higher order terms, e.g., nonlinear terms. Thus, models with more complex kinetic expressions may be easier to structurally identify compared to simpler models. To illustrate this, we use the framework presented in [79] on motif 3 for cases B7 and B8 shown in Fig 9. For case B7 we see that measurements *A* and *j*_*s*_ are insufficient for structural identifiability, and we find the following translational transformation:
  $$\begin{array}{c} \begin{array}{c} E(t)=-\epsilon +E(t)\\ k_{i}=\epsilon \cdot k_{c}+k_{i}. \end{array} \end{array}$$ (29)
  On the other hand, the same measurements are sufficient for structural identifiability for case B8, as shown in Fig 9. We find no symmetries for case B8, implying that the symmetry is broken by the more complex degradation of *E*.
- Following up on the notion of self–coupling, we note that introducing more complex kinetics by adding a new parameter, $K_{a}^{A}$ or $K_{a}^{E}$, going from first order to saturable activation kinetics (Eqs (5)–(8)), does not alter the identifiability score. This is seen by individually comparing the blue and orange cells in Table 1. Similarly, an increase of kinetic complexity from adding the parameter $K_{M}^{E}$, going from first order to Michaelis–Menten degradation of *E* (Eqs (10) and (11)), reveals the same result (compare the two brown cells in Table 1). These results illustrate how an increase in the number of parameters (expected to reduce the identifiability score) is compensated for by a corresponding increase in model complexity (increased self–coupling).

To summarize, our results indicate that an increase of parameters in a model may cause more instances to be structurally unidentifiable, whereas increased self–coupling may have the opposite effect. These results reveal that the two dimensions of complexity may affect structural identifiability in opposite ways. Increased model size appears negative, whereas more complex reaction kinetics appear positive. Thus, an increase of model size can be compensated for by more complex kinetic expressions if they increase self–coupling in a way that also improves structural identifiability.

#### Comparison of motifs

In order to compare motifs, we have in Table 2 organized the aggregated identifiability scores for each motif, and we consider the cases for one and two disturbances separately.

From the aggregated identifiability scores in Table 2 we note the following:

- Activating motifs with one disturbance have very high identifiability scores, regardless of the different expressions for the degration of *E* (white cells on row 3 and 4).
- The identifiability scores of inhibiting motifs (gray cells) with one or two disturbances depend heavily on the different expressions for the degration of *E*. A similar general result is only to a limited extent valid for the activating controllers (white cells).
- The identifiability scores for motifs with one disturbance are generally high, except for the inhibiting motifs with zero order degradation of *E* (gray cells in row 3 with an average score of 0.25).
- For motifs with two disturbances, outflow controllers in general have a higher average identifiability score than inflow controllers (row 6).
- The average scores for motifs 3 and 4 with two disturbances are 0.33 (row 5), distinctly lower than all the other motifs.

We find it noteworthy that outflow controllers have higher identifiability scores than inflow controllers for the cases with two disturbances. One possible reason is that the compensatory flow *j*_*c*_ for inflow controllers is a zero order synthesis reaction with respect to *A*, whereas it is a first order degradation reaction with respect to *A* for outflow controllers. Outflow controllers thus have increased self–coupling compared to inflow controllers.

### Analysis of structural unidentifiability

In this section we look into which system parameters/states are unidentifiable/unobservable for a selection of unidentifiable instances. We use the unidentifiable instances for motif 3 case B8 as an example, corresponding to the red and yellow cells in Fig 7. To find which system parameters/states that cause the overall model to be unidentifiable we use the STRIKE–GOLDD 4.0 MATLAB app [81], and the results are shown in Table 3. The rows are sorted with respect to the categories *Case and motif dependent* and *Always insufficient* along with the type of measurement, the rank, and the identifiable parameters. The main findings are summarized as:

- All structurally unidentifiable instances have unobservable states.
- Two measurements related to the same species rarely increase the number of identifiable parameters compared to a single measurement.
- Given the structure of motif 3, see Fig 1, and the system equations in Eqs (1) and (2), it is not straight forward to interpret the results for a single flow measurement. Especially the fact that the compensatory flow *j*_*c*_ provides limited information about system parameters, and no information about states. In contrast, the flow *d*_*o*_ provides much more information about both system parameters and states.

**Table 3:** Analysis of structural unidentifiability of motif 3 case B8.

	Measurement combinations	Rank	Identifiable parameters	Unidentifiable parameters	Observ. states	Unobserv. states
Measurements related to both *A* and *E*. *Case and motif dependent*	[*E*, *d*_*i*_] [*d*_*i*_, *j*_*s*_] [*d*_*i*_, *j*_*d*_]	7	[*k*_*i*_, *k*_*o*_, *k*_*d*_]	[*k*_*c*_, *k*_*s*_, $K_{i}^{A}$]	[*E*]	[*A*]
	[*E*, *j*_*c*_] [*j*_*c*_, *j*_*s*_] [*j*_*c*_, *j*_*d*_]	7	[*k*_*c*_, *k*_*o*_, *k*_*d*_]	[*k*_*i*_, *k*_*s*_, $K_{i}^{A}$]	[*E*]	[*A*]
Measurements related only to *A*. *Always insufficient*	[*A*], [*d*_*o*_] [*A*, *d*_*i*_], [*A*, *d*_*o*_] [*A*, *j*_*c*_], [*d*_*i*_, *d*_*o*_] [*d*_*i*_, *j*_*c*_], [*d*_*o*_, *j*_*c*_]	7	[*k*_*i*_, *k*_*o*_, *k*_*d*_, $K_{i}^{A}$]	[*k*_*c*_, *k*_*s*_]	[*A*]	[*E*]
	[*j*_*c*_]	6	[*k*_*o*_, *k*_*d*_]	[*k*_*i*_, *k*_*c*_, *k*_*s*_, $K_{i}^{A}$]	[-]	[*A*, *E*]
	[*d*_*i*_]	1	[*k*_*i*_]	[*k*_*o*_, *k*_*c*_, *k*_*s*_, *k*_*d*_, $K_{i}^{A}$]	[-]	[*A*, *E*]
Measurements related only to *E*. *Always insufficient*	[*E*], [*j*_*s*_], [*j*_*d*_] [*E*, *j*_*s*_] [*E*, *j*_*d*_] [*j*_*s*_, *j*_*d*_]	6	[*k*_*o*_, *k*_*d*_]	[*k*_*i*_, *k*_*c*_, *k*_*s*_, $K_{i}^{A}$]	[*E*]	[*A*]

Detailed analysis of the unidentifiable instances of motif 3 case B8. Results are organized with respect to the measurement combinations within the categories *Case and motif dependent* and *Always insufficient*, the rank, and the indentifiable parameters.

In supplementary material S1 File we have in addition analyzed motif 1 case B8, and we find simlar results. We expect the three main findings listed above to be universal across all motif and case platforms.

### Graphical visualization

The connections between measurements and identifiable/unidentifiable parameters in the motifs can be illustrated by directed graphs. As an example, we use motif 1 case B8 in Fig 7, where we restrict the presentation to the following list of measurement combinations involving *A*, *E*, *d*_*i*_ and *j*_*d*_:

- Structurally identifiable combinations of measurements related to both *A* and *E*, i.e., *A* or *d*_*i*_, and *E* or *j*_*d*_. These are shown in lines 3, 13, 14 and 22 in Fig 7.
- Structurally unidentifiable combinations of measurements related only to *A*, i.e., *A* and/or *d*_*i*_ (lines 1, 4 and 9).
- Structurally unidentifiable combinations of measurements related only to *E*, i.e., *E* and/or *j*_*d*_ (lines 2, 8 and 18).

The directed graphs of these combinations are shown in Fig 10. In order to include flow as measurement we used method 1, as the generalized flow measurement equation *y*(*t*) = *j*(*t*) is easier to graphically illustrate compared to a measurement equation from method 2. See supplementary S2 Text Fig a for model equations.

In accordance with our previous findings, the directed graphs illustrate that measurements related to both species are necessary to obtain structural identifiability. Also, we see that *k*_*c*_ is impossible to identify without measurements related to both species, similar to the results in Table 3 for motif 3. Moreover, comparing panels E and G, and also H and J, of Fig 10 show that an additional flow measurement related to the same species does not increase the number of identifiable parameters in this case. Another observation is that *k*_*o*_ is identifiable from almost all measurement combinations in panels E to J, and that neither *k*_*d*_ nor *k*_*s*_ is identifiable from measurements related to *A*. Measurement of *d*_*i*_ alone results in only *d*_*i*_ being observable, which is a reasonable result as *d*_*i*_ is a constant flow independent of any other variables in the model. Essentially, as *d*_*i*_(*t*) = *k*_*i*_, measuring *d*_*i*_ is the same as knowing the value of *k*_*i*_ a priori.

### Antithetic controller motifs

Results for the antithetic controller motifs are in line with our findings from the basic controller motifs. A selection of results are shown in Fig 11. Similarly to the basic motifs we have in the figure used different grayscale colors to indicate measurements related to species *A*, *E*_1_, and *E*_2_. The annihilation flow *j*_*a*_ is related to both controller species and is therefore marked with both grayscale colors.

As for the basic controller motifs, we classify the measurement combinations as either *Always insufficient*, *Always sufficient* or *Case and motif dependent*, summarized as:

- *Always insufficient*:
  – one concentration measurement.
  – one flow measurement.
  – one flow and one concentration measurement related to the same species.
  – two flow measurements related to the same species.
  – two measurements related only to the controller species *E*_1_ and *E*_2_.
- *Always sufficient*:
  – measurement of species concentration *A* and one other species concentration.
  – measurement of species concentration *A* and a flow related to another species.
  – measurement of the flow *d*_*o*_ and a concentration or flow related to one of the controller species.
- *Case and motif dependent*:
  – one flow measurement related to *A*, other than *d*_*o*_, and one concentration measurement related to another species.
  – two flow measurements, related to *A* and another species.

From a case to case comparison from Figs 2 and 4, the antithetic motifs show similar results as the basic motifs. Adding an unknown disturbance reduces the number of structurally identifiable instances, although the change is less prominent for the antithetic motifs than for the basic motifs. Similarly, a change from first order to saturable activation kinetics has no effect on structural identifiability, i.e., the left and right hand sides of Fig 11 are identical. The antithetic motifs 3 and 4 with two disturbances have significantly lower identifiability scores (0.36) than any of the other antithetic motifs, similar to the results for the basic motifs in Table 2.

### Comparison of basic and antithetic motifs

The basic and antithetic motifs have comparable model structures, where the main difference is the number of controller species. To achieve structural identifiability for the basic motifs, measurements related to both the controlled species *A* and the controller species *E* are necessary. The same requirement applies to the antithetic motifs, where one measurement related to *A* and one measurement related to either of the controller species, *E*_1_ or *E*_2_, are necessary for structural identifiability.

To compare results between basic and antithetic motifs we consider only cases with similar reaction kinetic assumptions. From the case descriptions shown in Figs 2 and 4, we have that cases B2, B5, B8, and B11 for the basic motifs are comparable to cases A1, A2, A3, and A4 for the antithetic motifs, respectively. Note that degradation of *E*_1_ and *E*_2_ for the antithetic motifs, the annihilation flow *j*_*a*_ is always first order with respect to *E*_1_ and with respect to *E*_2_, even though it is second–order overall. We disregard measurement combinations that are always insufficient for structural identifiability and calculate an average identifiability score of 0.90 for the basic motifs and 0.89 for the antithetic motifs (see supplementary S1 and S2 Files for calculations). We find this result somewhat surprising, as the antithetic motifs have three states and an average of 10 total states and parameters whereas the basic motifs have two states and an average of 8 total states and parameters. Based on results for the basic motifs we expected more instances to be structurally unidentifiable for the antithetic motifs because they have more parameters to identify with the same number of measurements. A possible explanation for this observation is the annihilation flow *j*_*a*_, which includes both *E*_1_ and *E*_2_ terms. Similarly to the definition of self–coupling describing how a state variable appears in its own state equation, we use the term *cross–coupling* to describe how a state variable appears in another state equation. Thus, increased cross–coupling appears to increase structural identifiability in much the same way as increased self–coupling.

## Discussion

We have investigated structural identifiability of basic and antithetic controller motifs with varying degree of model complexity. Investigating a total of 3648 instances of model structure and measurement combinations allowed us to quantitatively assess how two dimensions of model complexity, i.e., model size and complexity of reaction kinetic expressions, affected structural identifiability. Unsurprisingly, models of greater size are harder to structurally identify, as more parameters must be identified with the same number of measurements. Regarding the complexity of reaction kinetic expressions, our results indicated that both increased self–coupling and cross–coupling are beneficial for structural identifiability as they introduce more complexity and/or nonlinearity. However, given that more complex expressions may also introduce new parameters, which increases model size, more complex expressions may in some cases not benefit structural identifiability. Nevertheless, our results fit well with reported findings indicating that models with more complex kinetic expressions, e.g., in the form of nonlinearities, often are easier to structurally identify than simpler models [75–77]. Related to this topic, we have shown that Lie symmetries found in simpler controller motifs can be broken in more complex and/or nonlinear motifs [78–80].

We have shown that increasing the number of parameters in a model by including more flow expressions (i.e., disturbances), makes the model less likely to be structurally identifiable. On the other hand, if the increased number of parameters is accompanied by an increase in model complexity in the form of increased self–coupling or increased cross–coupling, structural identifiability may be unaffected. Specificallly, this implies that with the aim of achieving structural identifiability, it may be unnecessary to simplify Michaelis–Menten type expressions even though this reduces the number of parameters.

The reaction kinetic expressions investigated in this paper range from simple zero order degradation, to first order, and further to saturable kinetics. Although all of these reaction kinetics are widely used in the presentation of controller motifs [28, 50, 56], investigating structural identifiability of controller motifs with different and more complex reaction kinetics is certainly of interest for future studies. A noteworthy example is motifs with autocatalytic generation of *E* shown to provide perfect adaption in the presence of first order degradation of *E* [27, 70]. Another example is the use of higher order Hill kinetics between *A* and *E* [82], which can account for cooperative activation or inhibition [83].

A lack of structural identifiability is often associated with a high parameter–to–output ratio [9, 63]. This can be seen in the Goodwin oscillator model [11, 63, 84], the *β*IG model [85–87], and in our own results. To fix a model’s lack of structural identifiability there are mainly two approaches, i.e., reparametrization [88, 89] or to apply more model outputs/measurements [5, 6], where both will reduce the parameter–to–output ratio. Regarding the latter approach, the specific choice of which measurement to include is of great importance with respect to structural identifiability [16, 17]. In this context, we suggest to use flow measurements as alternative model outputs, and we have presented two different modeling approaches on how to incorporate flow measurements into a model. We have shown that for controller motifs, two measurements related to different chemical species are necessary for structural identifiability. This condition can be met using a combination of either two concentration measurements, one flow and one concentration measurement, or two flow measurements. Out of the 3648 instances analyzed, 1568 instances included measurements related to different chemical species, and among those instances 80% were structurally identifiable. Calculations are detailed in supplementary S1 and S2 Files.

In regards to estimating parameters in practice, time series measurements of the chosen outputs are necessary. This is often challenging to achieve in biological experiments because many methods of performing measurements, such as mass spectrometry, are highly invasive and may involve steps that kill the cells in the process. Thus, generating time series of measurements may become prohibitive because of associated costs. Flows may in some cases be easier to measure than internal concentrations, especially flows across the cell membrane, and several methods allow for real–time measurements of flows *in vivo* [90]. Although the practicality of obtaining experimental data is outside the scope of this paper, we will discuss some examples of how flow measurements can be used to provide time series measurements.

In an experimental setup there are typically different kinds of measurements available, e.g., internal and external concentrations together with possible flow measurements. Flow described as transport across a cell membrane can be estimated using measurements of only external concentrations, which are much easier to measure compared to internal concentrations. Several experimental methods also allow direct measurement of such trans–membrane flows. One example is microelectrode ion flux estimation (MIFE) [91, 92], where transport of ions such as H^+^, Ca^2+^, K^+^ and Na^+^ can be measured using ion–selective microelectrodes. Another example is fluorescence microphotolysis [93], which can measure both trans–membrane and intracellular transport. We have previously presented a model of Na^+^/K^+^ homeostasis in epithelial enterocytes [94] where Na,K–ATPase acts as an outflow controller similar to motif 5 in Fig 1. For this controller motif, both the inflow of Na^+^, corresponding to *d*_*i*_ in the general motif, and the Na,K–ATPase–activated outflow of Na^+^, corresponding to *j*_*c*_ in the general motif, could potentially be measured using the experimental methods described above.

Another approach is to measure intracellular flow indirectly through an easy to measure extracellular process. One example of such is measurement of glycolytic and oxidative ATP production based on extracellular flow measurements of oxygen consumption and extracellular acidification [95]. For a controller motif acting as part of the energy metabolism of the cell, both inflows and outflows are flow measurement candidates. In this context, we have previously analyzed structural identifiability of a cancer metabolism model [96]. It proved to be structurally identifiable with five measurements, i.e., three extracellular concentrations and two flow measurements of both oxygen consumption rate (OCR) and proton production rate (PPR, a measurement derived from extracellular acidification rate). Both of these flow measurements were performed using a Seahorse XF analyzer, and this experimental setup allows for in vivo time–series assays with dynamic responses to perturbations at a low cost.

Regarding how to incorporate flow measurements into a model we found that the two shown methods gave identical results for structural identifiability. Method 1 requires differentiation of flow expressions, which results in more complex models and longer execution times. Thus, method 2 is favorable in terms of computational cost and ease of implementation. Method 1 is more suitable for a graphical presentation as shown in Fig 10. When analyzing structural identifiability results for models as a whole, there is no difference between the two methods. Both methods have advantages in different situations, although method 2 will likely be preferred because of faster computations.

Concerning our chosen method to investigate structural identifiability, symbolic methods are known to be computationally demanding, especially in terms of memory requirements. The most demanding task in the computations is the symbolic rank calculation of the $O_{I}\left(\tilde{x}\right)$ matrix in Eq (22). We found that execution times showed approximately exponential growth for increasing model size, and in line with previous findings [63] we reached a limit of what was practically feasible for models with 10–11 parameters. Although numerical rank calculations provided consistent results where symbolic calculations failed, there is likely a limit to what is practically feasible also for numerical rank calculations using this method. However, for rational models there exist numerical algorithms that can handle models with more parameters and that are more computationally efficient [58, 81, 97]. In recent years, several methods for structural identifiability analysis of more general models with many states and parameters have also been presented. One approach is to use a hybrid numerical and symbolic method where numerical simulations are used to find likely candidates for structurally unidentifiable parameters [62, 98]. A symbolic calculation with the reduced set of assumed structurally unidentifiable parameters can then be performed for confirmation. Another approach is to decompose a model into smaller submodels which can be analyzed using symbolic methods [63].

## Supporting information

S1 Text Structured literature search. Search strategy and results.
(PDF)

S2 Text Motif overview. Extended overview of model equations for all motifs and cases and examples of flows other than *j*_*c*_ being used as model output.
(PDF)

S1 File Basic motif results. Raw data results and calculations for all motifs, cases and methods for the basic controller motifs.
(XLSX)

S2 File Antithetic motif results. Raw data results and calculations for all motifs, cases and methods for the antithetic controller motifs.
(XLSX)

S3 File Code. Zip file containing MATLAB code used in the calculations. MATLAB is required to execute the code, but .m files can be opened as pure text files.
(ZIP)
