# Supplementary material for: Structural identifiability of biomolecular controller motifs with and without flow measurements as model output
Source: PLoS Comput Biol. 2023 Aug 28;19(8):e1011398. doi: 10.1371/journal.pcbi.1011398 (PMC10491402; doi:10.1371/journal.pcbi.1011398)
Supplement: S1 Text — Search strategy and results. (PDF) [file pcbi.1011398.s001.pdf]

Structured literature search

Databases used in the search:

- PubMed – Biomedical research
- Scopus – Large multidisciplinary database (Elsevier)
- Web of Science – Large multidisciplinary database (Clarivate)

Paper title: Structural identifiability of biomolecular controller motifs with and without flow measurements as model output

Three main research questions/topics:

1. Investigate structural identifiability of controller motifs.
2. Investigate how flow measurements compares to concentration measurements as model outputs for biological models.
3. Structural identifiability when flow measurements are used instead of, or in combination with, concentration measurements.

For each topic we read through the abstracts of papers that showed up in the search to find relevant papers. If the number of results surpassed 100 entries, we read through the abstracts of the first 100 entries from each database.

Topic 1: Investigate the structural identifiability of controller motifs

We have performed two searches on this topic.

- A narrow search regarding only controller motifs to find if anyone has investigated structural identifiability of controller motifs before.
- A wider search of structural identifiability of biological models to find relevant literature and detect any possible entries missed by the first search

Narrow search to find whether this has been investigated before

Concept 1: Structural identifiability

Keywords:

- Structural identifiability
- Identifiability

PubMed search string: "structural* identifiab*" OR identifiability

Scopus search string: (structural* W/2 identifiab*) OR identifiability

Web of Science search string: (Structural* NEAR/2 identifiab*) OR identifiability

Concept 2: Controller motifs

Keywords:

- Controller motifs
- Antithetic control
- Antithetic feedback
- Homeostatic control

PubMed search string: "control* motif*" OR (antithetic AND control*) OR (antithetic AND feedback) OR "homeostatic control*"

Scopus search string (Article title, Abstract, Keywords): (control* W/2 motif*) OR (antithetic W/5 control*) OR (antithetic AND feedback) OR (homeostatic W/5 control*)

Web of Science search string (Topic: Searches title, abstract, author keywords, and Keywords Plus.): (control* NEAR/2 motif*) OR (antithetic NEAR/5 control*) OR (antithetic NEAR/5 feedback) OR (homeostatic NEAR/5 control*)

Search	PubMed	Scopus	Web of Science
Concept 1	1783	8737	7039
Concept 2	1781	4107	3295
Concept 1 AND 2	0	2	1

The search using concepts 1 and 2 found in total three articles. However, we found that none of these investigated structural identifiability of controller motifs.

Wide search to find relevant literature

Concept 1: Structural identifiability

Keywords and search strings as previously described.

Concept 2: Biological models

Keywords:

- Systems biology
- Biological model/circuit/network
- Biomolecular model/circuit/network
- Cell model/circuit/network
- Kinetic model/circuit/network
- Biochemical model/circuit/network
- Physiological model/circuit/network
- Genetic model/circuit/network

Applicable MeSH terms (PubMed):

- "Models, Biological"[MeSH]
- "Feedback, Physiological"[MeSh]
- "Kinetics"[MeSh]
- "Systems Biology"[MeSh]

PubMed search string: "systems biology" OR

"biological model*" OR "biomolecular model*" OR "cell model*" OR "kinetic model*" OR "biochemical model*" OR "physiological model*" OR "genetic model*" OR

"biological circuit*" OR "biomolecular circuit*" OR "cell circuit*" OR "kinetic circuit*" OR "biochemical circuit*" OR "physiological circuit*" OR "genetic circuit" OR

"biological network*" OR "biomolecular network*" OR "cell network*" OR "kinetic network*" OR "biochemical network*" OR "physiological network*" OR "genetic network" OR

"Models, Biological"[MeSH] OR "Feedback, Physiological"[Mesh] OR "Kinetics"[Mesh] OR "Systems Biology"[Mesh]

Scopus search string: “systems biology” OR (biological W/4 model*) OR (biomolecular W/4 model*) OR (cell W/4 model*) OR (kinetic W/4 model*) OR (biochemical W/4 model*) OR (physiological W/4 model*) OR “genetic W/4 model*”

OR (biological W/4 circuit*) OR (biomolecular W/4 circuit*) OR (cell W/4 circuit*) OR (kinetic W/4 circuit*) OR (biochemical W/4 circuit*) OR (physiological W/4 circuit*) OR “genetic W/4 circuit*”

OR (biological W/4 network*) OR (biomolecular W/4 network*) OR (cell W/4 network*) OR (kinetic W/4 network*) OR (biochemical W/4 network*) OR (physiological W/4 network*) OR “genetic W/4 network*”

Web of Science search string: “systems biology” OR

(biological NEAR/4 model*) OR (biomolecular NEAR /4 model*) OR (cell NEAR /4 model*) OR (kinetic NEAR /4 model*) OR (biochemical NEAR /4 model*) OR (physiological NEAR /4 model*) OR (genetic NEAR/4 model*)

OR (biological NEAR/4 circuit*) OR (biomolecular NEAR /4 circuit*) OR (cell NEAR /4 circuit*) OR (kinetic NEAR /4 circuit*) OR (biochemical NEAR /4 circuit*) OR (physiological NEAR /4 circuit*) OR (genetic NEAR/4 circuit*)

OR (biological NEAR/4 network*) OR (biomolecular NEAR /4 network*) OR (cell NEAR /4 network*) OR (kinetic NEAR /4 network*) OR (biochemical NEAR /4 network*) OR (physiological NEAR /4 network*) OR (genetic NEAR/4 network*)

Search	PubMed	Scopus	Web of Science
Concept 1	1783	8737	7039
Concept 2	1 479 866	949 744	115864
Concept 1 AND 2	729	993	327

Topic 2: How does flow measurements as measured outputs compare to concentration measurements for biological models

Concept 1: Biological models

Keywords and search strings as previously described.

Concept 2: Flow/flux measurements

Keywords:

- Flow measurement
- Flux measurement
- Rate measurement
- Reaction measurement

Applicable MeSH terms (PubMed):

- "Metabolic Flux Analysis"[MeSh]

PubMed search string: “flow measur*” OR “flux measur*” OR “rate measur*” OR “reaction measur*” or "Metabolic Flux Analysis"[Mesh]

Scopus search string: (flow W/1 measur*) OR (flux W/1 measur*) OR (rate W/1 measur*) OR (reaction W/1 measur*)

Web of Science search string: (flow NEAR/1 measur*) OR (flux NEAR/1 measur*) OR (rate NEAR/1 measur*) OR (reaction NEAR/1 measur*)

Concept 3: Model output

Keywords:

- Measured output
- Model output
- System output
- Output function

PubMed search string: "measured output*" OR "model output*" OR "system output*" OR "output function*"

Scopus search string: (measured W/1 output*) OR (model W/1 output*) OR (system W/1 output*) OR (output W/1 function*)

Web of Science search string: (measured NEAR/4 output*) OR (model NEAR/4 output*) OR (system NEAR/4 output*) OR (output NEAR/4 function*)

Search string	PubMed	Scopus	Web of Science
Concept 1	1 479 866	949 744	115864
Concept 2	17 901	237 208	125308
Concept 3	4225	97323	107722
Concept 1 AND 2	2346	5247	227
Concept 1 AND Concept 2 AND Concept 3	3	27	1

Topic 3: Structural identifiability when flow measurements are used as model output

Concept 1: Structural identifiability

Keywords and search strings as previously described.

Concept 2: Flow/flux measurements

Keywords and search strings as previously described.

Concept 3: Model output

Keywords and search strings as previously described.

Search string	PubMed	Scopus	Web of Science
Concept 1	1783	8737	7039
Concept 2	17 901	237 208	125308
Concept 3	4225	97323	107722
Concept 1 AND 2	7	38	32
Concept 1 AND Concept 2 AND Concept 3	0	0	0

For topics 2 and 3 we did not find any relevant articles comparing flow and concentration measurements as model output with regards to structural identifiability.
