# Supplementary material for: Structural identifiability of biomolecular controller motifs with and without flow measurements as model output
Source: PLoS Comput Biol. 2023 Aug 28;19(8):e1011398. doi: 10.1371/journal.pcbi.1011398 (PMC10491402; doi:10.1371/journal.pcbi.1011398)
Supplement: S2 Text — Extended overview of model equations for all motifs and cases and examples of flows other than jc being used as model output. (PDF) [file pcbi.1011398.s002.pdf]

S2 Text - Motif overview

This supplementary shows additional examples where flows are used as ouput in controller motifs along with complete system equations for the basic and antithetic controller motifs. The supplementary is divided into three sections:

1. **Examples of flows used as output.** Three additional examples of motifs where flows are used as output.

2. **Basic controller motifs.** Complete overview of system equations for the basic motifs for all cases investigated (cases B1-B12, see Fig d).

3. **Antithetic controller motifs.** Complete overview of system equations for the antithetic motifs for all cases investigated (cases A1-A4, see Fig e).

1 Examples of flows used as output

In this section of the supplementary text we use three different motifs/cases to illustrate the use of other flows than $j_c(t)$ as measurements, i.e., $d_i(t)$, $j_d(t)$, $j_s(t)$, and $j_d(t)$. For each motif, we use a combination of two out of these four flows as model output.

The first motif shown in Fig a is motif 1/case B8 related to panel D in Fig 10 in the main paper, where we use $d_i(t)$ and $j_d(t)$ as model output.

The second motif shown in Fig b is motif 3/case B8 related to the first row of Table 3 in the main paper, where we use $d_i(t)$ and $j_s(t)$ as model output.

The last motif shown in Fig c is motif 6/case B12 related to the lower right subpanel of Fig 9 in the main paper, where we use $d_o(t)$ and $j_d(t)$ as model output (corresponds to the third last row of the subpanel).

A

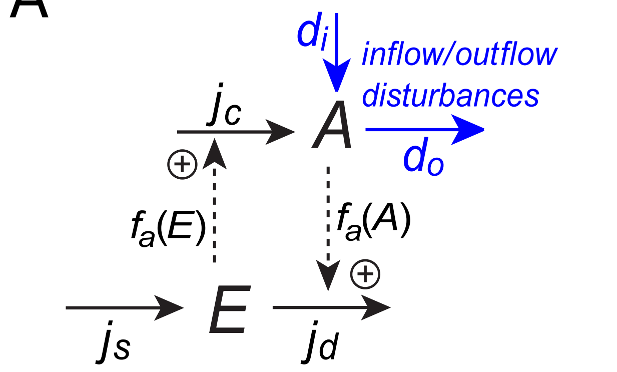

B

$$d_i(t) = k_i$$
$$d_o(t) = k_o \cdot A(t)$$
$$j_c(t) = k_c \cdot E(t)$$
$$j_s(t) = k_s$$
$$j_d(t) = k_d \cdot A(t) \cdot E(t)$$

C

$$\frac{dA(t)}{dt} = d_i(t) - k_o \cdot A(t) + k_c \cdot E(t)$$
$$\frac{dE(t)}{dt} = k_s - j_d(t)$$
$$\frac{dd_i(t)}{dt} = 0$$
$$\frac{dj_d(t)}{dt} = \frac{j_d(t) \cdot (d_i(t) - k_o \cdot A(t) + k_c \cdot E(t))}{E(t) \cdot (A(t) - j_d(t) \cdot (j_d(t) - k_s))}$$
$$y(t) = [d_i(t),\ j_d(t)]$$
$$\tilde{x} = [A, E, {\color{red}d_i}, {\color{red}j_d}, k_o, k_c, k_s]$$

D

$$\frac{dA(t)}{dt} = k_i - k_o \cdot A(t) + k_c \cdot E(t)$$
$$\frac{dE(t)}{dt} = k_s - k_d \cdot A(t) \cdot E(t)$$
$$y(t) = [k_i,\ k_d \cdot A(t) \cdot E(t)]$$
$$\tilde{x} = [A, E, {\color{red}k_i}, k_o, k_c, k_s, {\color{red}k_d}]$$

Fig a. Motif 1 case B8. Panels A and B: Schematic of basic controller motif 1 and the corresponding flow expressions for case B8. Panels C and D: Method 1 and method 2, respectively, for including flow measurements as model output, showing state equations, model output and $\tilde{x}$. The total number of states and parameters to be identified (number of elements in $\tilde{x}$) is equal for both methods, though where method 1 has the states, d_i and j_d, method 2 has the original system parameters, k_i and k_d (all marked in red).

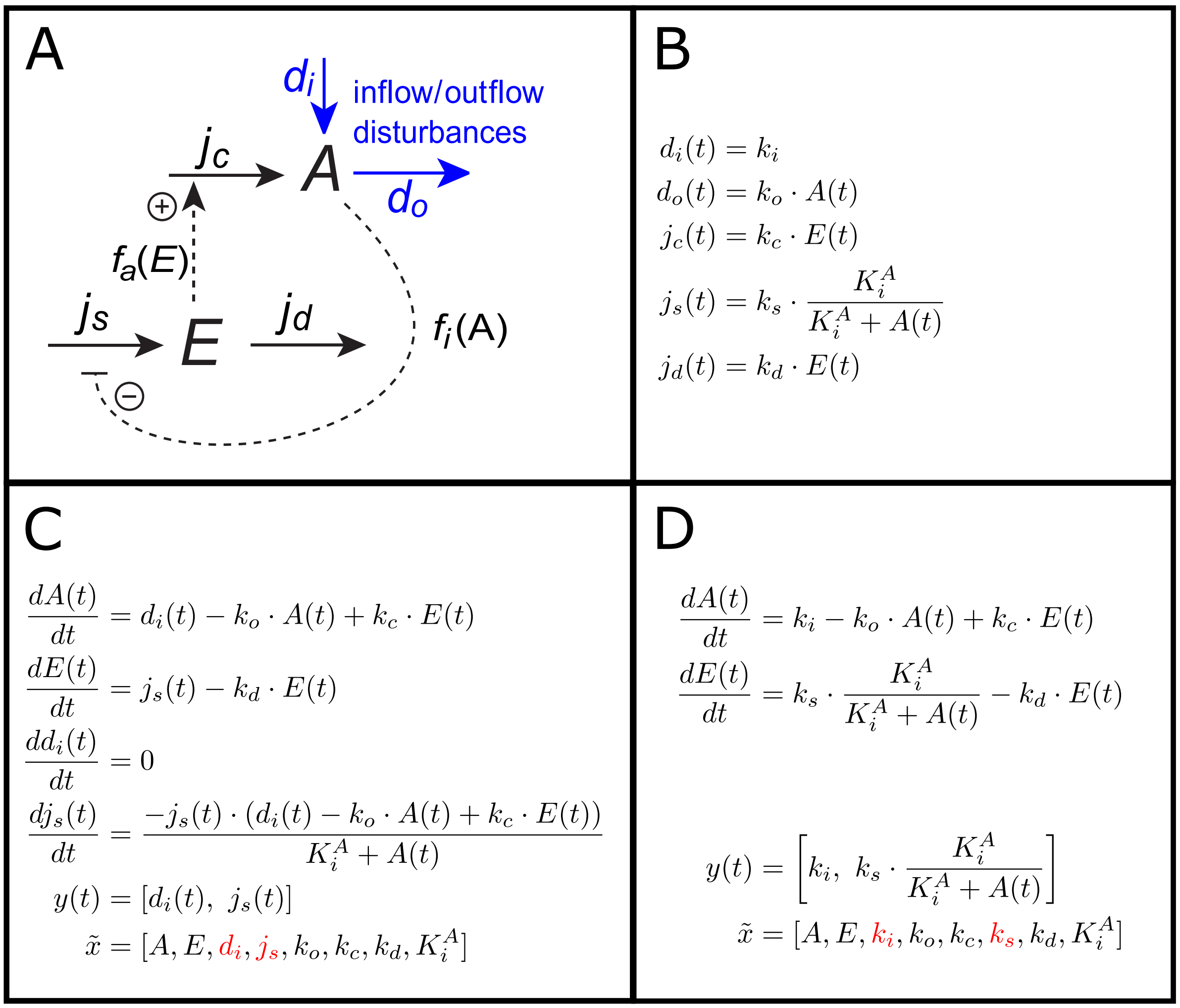

B

$$d_i(t) = k_i$$
$$d_o(t) = k_o \cdot A(t)$$
$$j_c(t) = k_c \cdot E(t)$$
$$j_s(t) = k_s \cdot \frac{K_i^A}{K_i^A + A(t)}$$
$$j_d(t) = k_d \cdot E(t)$$

C

$$\frac{dA(t)}{dt} = d_i(t) - k_o \cdot A(t) + k_c \cdot E(t)$$
$$\frac{dE(t)}{dt} = j_s(t) - k_d \cdot E(t)$$
$$\frac{dd_i(t)}{dt} = 0$$
$$\frac{dj_s(t)}{dt} = \frac{-j_s(t) \cdot (d_i(t) - k_o \cdot A(t) + k_c \cdot E(t))}{K_i^A + A(t)}$$
$$y(t) = [d_i(t),\ j_s(t)]$$
$$\tilde{x} = [A, E, d_i, j_s, k_o, k_c, k_d, K_i^A]$$

D

$$\frac{dA(t)}{dt} = k_i - k_o \cdot A(t) + k_c \cdot E(t)$$
$$\frac{dE(t)}{dt} = k_s \cdot \frac{K_i^A}{K_i^A + A(t)} - k_d \cdot E(t)$$
$$y(t) = \left[k_i,\ k_s \cdot \frac{K_i^A}{K_i^A + A(t)}\right]$$
$$\tilde{x} = [A, E, k_i, k_o, k_c, k_s, k_d, K_i^A]$$

Fig b. Motif 3 case B8. Panels A and B: Schematic of basic controller motif 3 and the corresponding flow expressions for case B8. Panels C and D: Method 1 and method 2, respectively, for including flow measurements as model output, showing state equations, model output and $\tilde{x}$. The total number of states and parameters to be identified (number of elements in $\tilde{x}$) is equal for both methods, though where method 1 has the states, d_i and j_s, method 2 has the original system parameters, k_i and k_s (all marked in red).

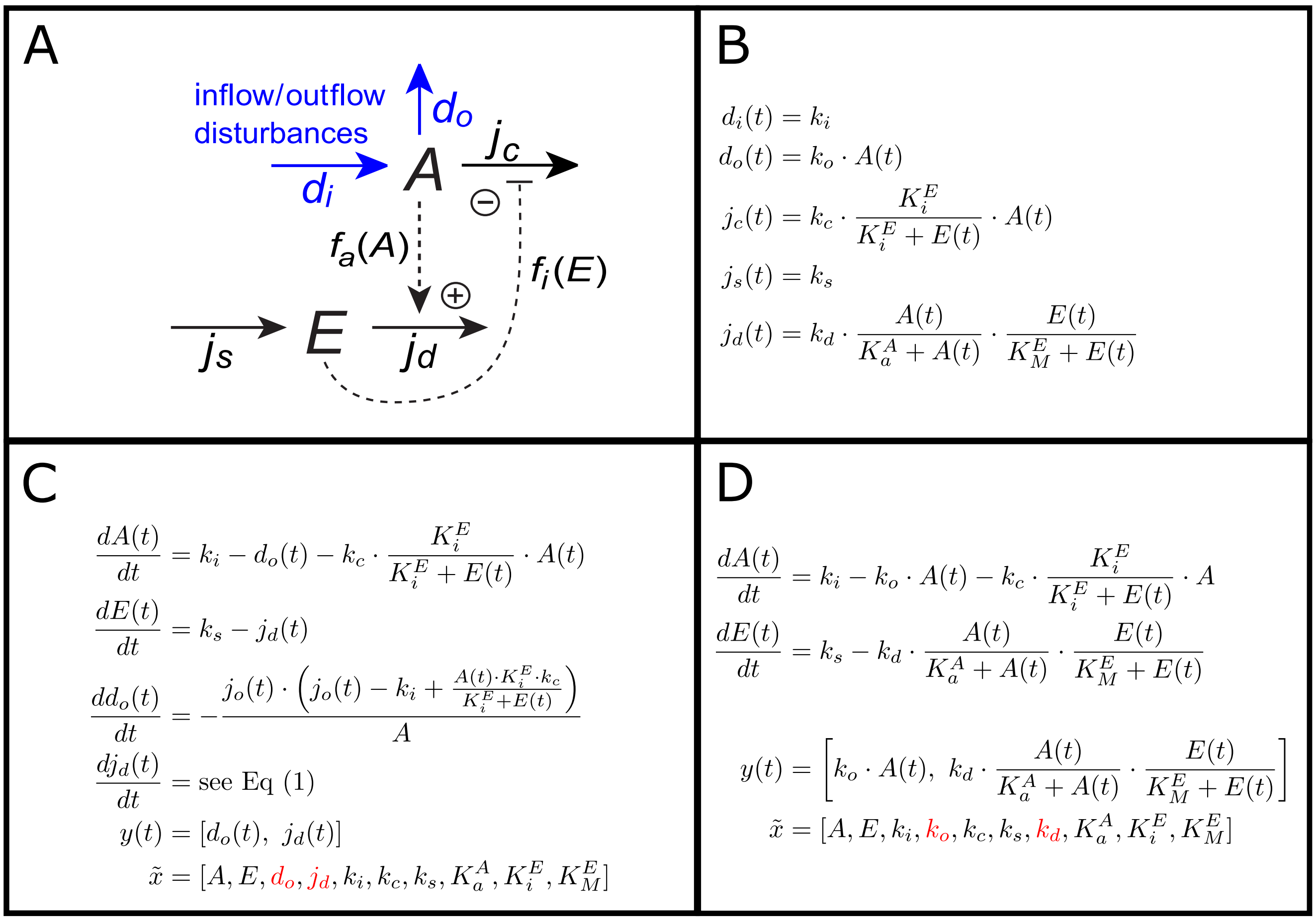

B

$$d_i(t) = k_i$$
$$d_o(t) = k_o \cdot A(t)$$
$$j_c(t) = k_c \cdot \frac{K_i^E}{K_i^E + E(t)} \cdot A(t)$$
$$j_s(t) = k_s$$
$$j_d(t) = k_d \cdot \frac{A(t)}{K_a^A + A(t)} \cdot \frac{E(t)}{K_M^E + E(t)}$$

C

$$\frac{dA(t)}{dt} = k_i - d_o(t) - k_c \cdot \frac{K_i^E}{K_i^E + E(t)} \cdot A(t)$$
$$\frac{dE(t)}{dt} = k_s - j_d(t)$$
$$\frac{dd_o(t)}{dt} = -\frac{j_o(t) \cdot \left(j_o(t) - k_i + \frac{A(t) \cdot K_i^E \cdot k_c}{K_i^E + E(t)}\right)}{A}$$
$$\frac{dj_d(t)}{dt} = \text{see Eq (1)}$$
$$y(t) = [d_o(t),\ j_d(t)]$$
$$\tilde{x} = [A, E, d_o, j_d, k_i, k_c, k_s, K_a^A, K_i^E, K_M^E]$$

D

$$\frac{dA(t)}{dt} = k_i - k_o \cdot A(t) - k_c \cdot \frac{K_i^E}{K_i^E + E(t)} \cdot A$$
$$\frac{dE(t)}{dt} = k_s - k_d \cdot \frac{A(t)}{K_a^A + A(t)} \cdot \frac{E(t)}{K_M^E + E(t)}$$
$$y(t) = \left[k_o \cdot A(t),\ k_d \cdot \frac{A(t)}{K_a^A + A(t)} \cdot \frac{E(t)}{K_M^E + E(t)}\right]$$
$$\tilde{x} = [A, E, k_i, k_o, k_c, k_s, k_d, K_a^A, K_i^E, K_M^E]$$

Fig c. Motif 6 case B12. Panels A and B: Schematic of basic controller motif 6 and the corresponding flow expressions for case B12. Panels C and D: Method 1 and method 2, respectively, for including flow measurements as model output, showing state equations, model output and $\tilde{x}$. The total number of states and parameters to be identified (number of elements in $\tilde{x}$) is equal for both methods, though where method 1 has the states, d_o and j_d, method 2 has the original system parameters, k_o and k_d (all marked in red).

$$\frac{dj_d(t)}{dt} = \frac{-K_a^A \cdot j_d(t) \cdot \left(d_o(t) - k_i + \frac{A(t) \cdot K_i^E \cdot k_c}{K_i^E + E(t)}\right)}{A(t) \cdot (K_a^A + A(t))} - \frac{K_M^E \cdot j_d(t) \cdot (j_d(t) - k_s)}{E(t) \cdot (K_M^E + E(t))} \tag{1}$$

2 Basic controller motifs

In this section we show the complete set of system equations for the basic controller motifs shown in Fig 1 in the main paper. The equations are organized into tables for each motif where we include the general state equations, the schematics of each motif, and the detailed expressions for $j_c(t)$, $j_s(t)$, and $j_d(t)$ for all cases. The inflow and outflow disturbances $d_i(t)$ and $d_o(t)$, shown in Eqs (3) and (4) in the main paper, are not included in the tables because they are unchanged between different motifs and cases. Disturbances marked in red in state equations and figure schematics are not included in the model for cases with one disturbance (cases B1–B6). Fig 2 from main paper showing case descriptions is reposted here as Fig d for convenience.

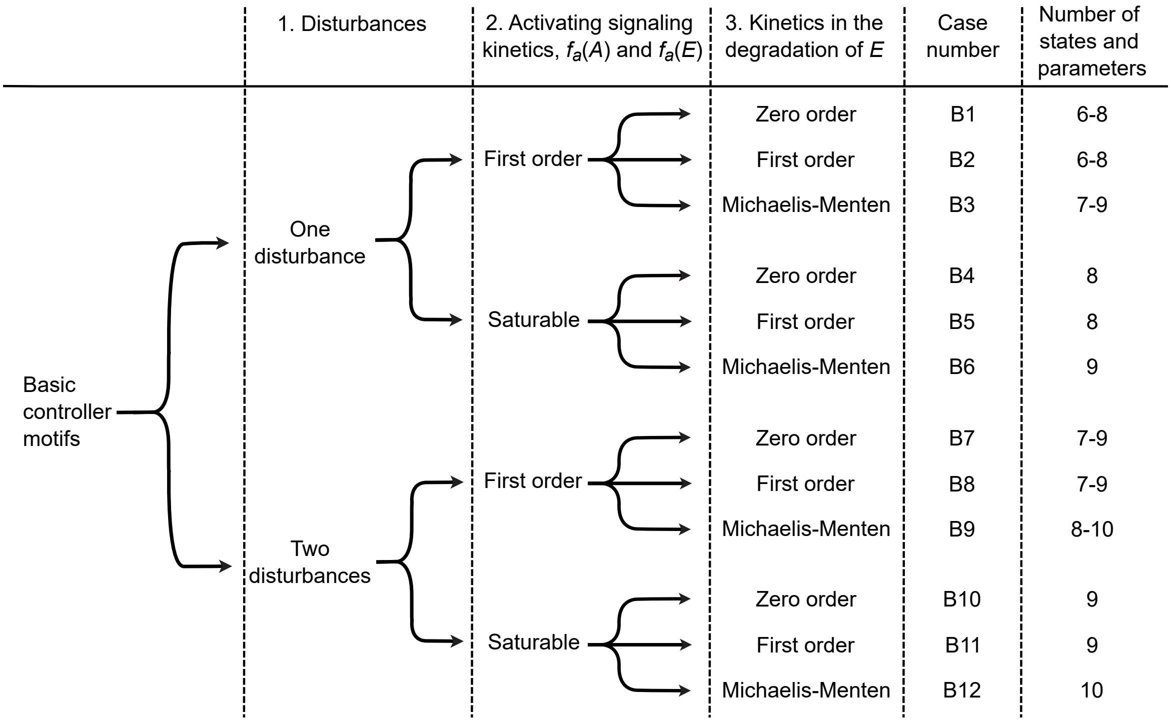

Fig d. Investigated cases for the basic motifs. Organization of the investigated cases for the basic controller motifs, taking into account the number of disturbances, the different expression candidates for the activation kinetics $f_a(A)$ and $f_a(E)$, and the expression candidates for the degradation kinetics of the controller species E. The combinations of these model complexities result in 12 cases named B1-B12, where the number of model parameters varies between 6 and 10.

**Motif 1**		First order activation kinetics	Saturable activation kinetics
State equations		Case B1/B7	Case B4/B10
$\frac{dA(t)}{dt} = d_i(t) - d_o(t) + j_c(t)$<br>$\frac{dE(t)}{dt} = j_s(t) - j_d(t)$	Zero order degradation of E	$j_c = k_c \cdot E$<br>$j_s = k_s$<br>$j_d = k_d \cdot A$	$j_c = k_c \cdot \frac{E}{K_a^E + E}$<br>$j_s = k_s$<br>$j_d = k_d \cdot \frac{A}{K_a^A + A}$
Motif schematic		Case B2/B8	Case B5/B11
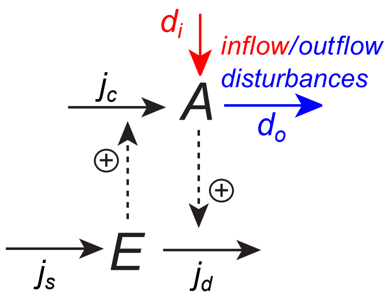	First order degradation of E	$j_c = k_c \cdot E$<br>$j_s = k_s$<br>$j_d = k_d \cdot A \cdot E$	$j_c = k_c \cdot \frac{E}{K_a^E + E}$<br>$j_s = k_s$<br>$j_d = k_d \frac{A}{K_a^A + A} \cdot E$
		Case B3/B9	Case B6/B12
	MM degradation of E	$j_c = k_c \cdot E$<br>$j_s = k_s$<br>$j_d = k_d \cdot A \cdot \frac{E}{K_M^E + E}$	$j_c = k_c \cdot \frac{E}{K_a^E + E}$<br>$j_s = k_s$<br>$j_d = k_d \cdot \frac{A}{K_a^A + A} \cdot \frac{E}{K_M^E + E}$

**Motif 2**		First order activation kinetics	Saturable activation kinetics
State equations		Case B1/B7	Case B4/B10
$\frac{dA(t)}{dt} = d_i(t) - d_o(t) + j_c(t)$<br>$\frac{dE(t)}{dt} = j_s(t) - j_d(t)$	Zero order degradation of E	$j_c = k_c \cdot \frac{K_i^E}{K_i^E + E}$<br>$j_s = k_s \cdot A$<br>$j_d = k_d$	$j_c = k_c \cdot \frac{K_i^E}{K_i^E + E}$<br>$j_s = k_s \cdot \frac{A}{K_a^A + A}$<br>$j_d = k_d$
Motif schematic		Case B2/B8	Case B5/B11
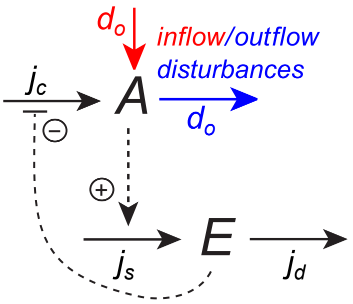	First order degradation of E	$j_c = k_c \cdot \frac{K_i^E}{K_i^E + E}$<br>$j_s = k_s \cdot A$<br>$j_d = k_d \cdot E$	$j_c = k_c \cdot \frac{K_i^E}{K_i^E + E}$<br>$j_s = k_s \cdot \frac{A}{K_a^A + A}$<br>$j_d = k_d \cdot E$
		Case B3/B9	Case B6/B12
	MM degradation of E	$j_c = k_c \cdot \frac{K_i^E}{K_i^E + E}$<br>$j_s = k_s \cdot A$<br>$j_d = k_d \cdot \frac{E}{K_M^E + E}$	$j_c = k_c \cdot \frac{K_i^E}{K_i^E + E}$<br>$j_s = k_s \cdot \frac{A}{K_a^A + A}$<br>$j_d = k_d \cdot \frac{E}{K_M^E + E}$

**Motif 3**		First order activation kinetics	Saturable activation kinetics
State equations		Case B1/B7	Case B4/B10
$\frac{dA(t)}{dt} = d_i(t) - d_o(t) + j_c(t)$ $\frac{dE(t)}{dt} = j_s(t) - j_d(t)$	Zero order degradation of E	$j_c = k_c \cdot E$ $j_s = k_s \cdot \frac{K_i^A}{K_i^A + A}$ $j_d = k_d$	$j_c = k_c \cdot \frac{E}{K_a^E + E}$ $j_s = k_s \cdot \frac{K_i^A}{K_i^A + A}$ $j_d = k_d$
Motif schematic		Case B2/B8	Case B5/B11
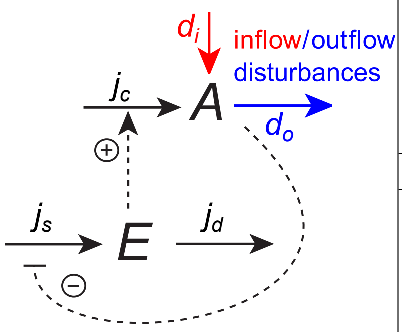	First order degradation of E	$j_c = k_c \cdot E$ $j_s = k_s \cdot \frac{K_i^A}{K_i^A + A}$ $j_d = k_d \cdot E$	$j_c = k_c \cdot \frac{E}{K_a^E + E}$ $j_s = k_s \cdot \frac{K_i^A}{K_i^A + A}$ $j_d = k_d \cdot E$
		Case B3/B9	Case B6/B12
	MM degradation of E	$j_c = k_c \cdot E$ $j_s = k_s \cdot \frac{K_i^A}{K_i^A + A}$ $j_d = k_d \cdot \frac{E}{K_M^E + E}$	$j_c = k_c \cdot \frac{E}{K_a^E + E}$ $j_s = k_s \cdot \frac{K_i^A}{K_i^A + A}$ $j_d = k_d \cdot \frac{E}{K_M^E + E}$

**Motif 4**		First order activation kinetics	Saturable activation kinetics
State equations		Case B1/B7	Case B4/B10
$\frac{dA(t)}{dt} = d_i(t) - d_o(t) + j_c(t)$ $\frac{dE(t)}{dt} = j_s(t) - j_d(t)$	Zero order degradation of E	$j_c = k_c \cdot \frac{K_i^E}{K_i^E + E}$ $j_s = k_s$ $j_d = k_d \cdot \frac{K_i^A}{K_i^A + A}$	$j_c = k_c \cdot \frac{K_i^E}{K_i^E + E}$ $j_s = k_s$ $j_d = k_d \cdot \frac{K_i^A}{K_i^A + A}$
Motif schematic		Case B2/B8	Case B5/B11
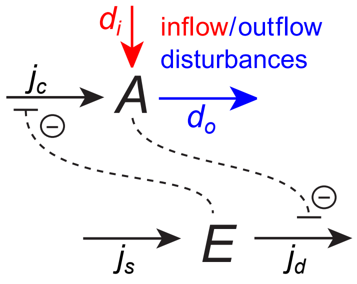	First order degradation of E	$j_c = k_c \cdot \frac{K_i^E}{K_i^E + E}$ $j_s = k_s$ $j_d = k_d \cdot \frac{K_i^A}{K_i^A + A} \cdot E$	$j_c = k_c \cdot \frac{K_i^E}{K_i^E + E}$ $j_s = k_s$ $j_d = k_d \cdot \frac{K_i^A}{K_i^A + A} \cdot E$
		Case B3/B9	Case B6/B12
	MM degradation of E	$j_c = k_c \cdot \frac{K_i^E}{K_i^E + E}$ $j_s = k_s$ $j_d = k_d \cdot \frac{K_i^A}{K_i^A + A} \cdot \frac{E}{K_M^E + E}$	$j_c = k_c \cdot \frac{K_i^E}{K_i^E + E}$ $j_s = k_s$ $j_d = k_d \cdot \frac{K_i^A}{K_i^A + A} \cdot \frac{E}{K_M^E + E}$

**Motif 5**		First order activation kinetics	Saturable activation kinetics
State equations		Case B1/B7	Case B4/B10
$\frac{dA(t)}{dt} = d_i(t) - d_o(t) - j_c(t)$<br>$\frac{dE(t)}{dt} = j_s(t) - j_d(t)$	Zero order degradation of E	$j_c = k_c \cdot E \cdot A$<br>$j_s = k_s \cdot A$<br>$j_d = k_d$	$j_c = k_c \cdot \frac{E}{K_a^E + E} \cdot A$<br>$j_s = k_s \cdot \frac{A}{K_a^A + A}$<br>$j_d = k_d$
Motif schematic		Case B2/B8	Case B5/B11
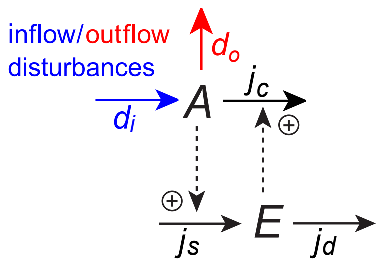	First order degradation of E	$j_c = k_c \cdot E \cdot A$<br>$j_s = k_s \cdot A$<br>$j_d = k_d \cdot E$	$j_c = k_c \cdot \frac{E}{K_a^E + E} \cdot A$<br>$j_s = k_s \cdot \frac{A}{K_a^A + A}$<br>$j_d = k_d \cdot E$
		Case B3/B9	Case B6/B12
	MM degradation of E	$j_c = k_c \cdot E \cdot A$<br>$j_s = k_s \cdot A$<br>$j_d = k_d \cdot \frac{E}{K_M^E + E}$	$j_c = k_c \cdot \frac{E}{K_a^E + E} \cdot A$<br>$j_s = k_s \cdot \frac{A}{K_a^A + A}$<br>$j_d = k_d \cdot \frac{E}{K_M^E + E}$

**Motif 6**		First order activation kinetics	Saturable activation kinetics
State equations		Case B1/B7	Case B4/B10
$\frac{dA(t)}{dt} = d_i(t) - d_o(t) - j_c(t)$<br>$\frac{dE(t)}{dt} = j_s(t) - j_d(t)$	Zero order degradation of E	$j_c = k_c \cdot \frac{K_i^E}{K_i^E + E} \cdot A$<br>$j_s = k_s$<br>$j_d = k_d \cdot A$	$j_c = k_c \cdot \frac{K_i^E}{K_i^E + E} \cdot A$<br>$j_s = k_s$<br>$j_d = k_d \cdot \frac{A}{K_a^A + A}$
Motif schematic		Case B2/B8	Case B5/B11
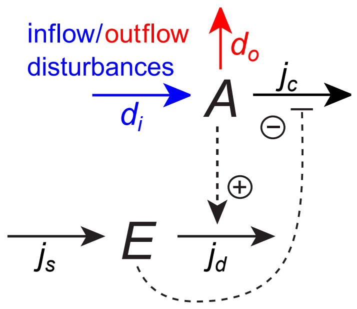	First order degradation of E	$j_c = k_c \cdot \frac{K_i^E}{K_i^E + E} \cdot A$<br>$j_s = k_s$<br>$j_d = k_d \cdot A \cdot E$	$j_c = k_c \cdot \frac{K_i^E}{K_i^E + E} \cdot A$<br>$j_s = k_s$<br>$j_d = k_d \cdot \frac{A}{K_a^A + A} \cdot E$
		Case B3/B9	Case B6/B12
	MM degradation of E	$j_c = k_c \cdot \frac{K_i^E}{K_i^E + E} \cdot A$<br>$j_s = k_s$<br>$j_d = k_d \cdot A \cdot \frac{E}{K_M^E + E}$	$j_c = k_c \cdot \frac{K_i^E}{K_i^E + E} \cdot A$<br>$j_s = k_s$<br>$j_d = k_d \cdot \frac{A}{K_a^A + A} \cdot \frac{E}{K_M^E + E}$

Motif 7		First order activation kinetics	Saturable activation kinetics
State equations		Case B1/B7	Case B4/B10
$\frac{dA(t)}{dt} = d_i(t) - d_o(t) - j_c(t)$<br>$\frac{dE(t)}{dt} = j_s(t) - j_d(t)$	Zero order degradation of E	$j_c = k_c \cdot E \cdot A$<br>$j_s = k_s$<br>$j_d = k_d \cdot \frac{K_i^A}{K_i^A + A}$	$j_c = k_c \cdot \frac{E}{K_a^E + E} \cdot A$<br>$j_s = k_s$<br>$j_d = k_d \cdot \frac{K_i^A}{K_i^A + A}$
Motif schematic		Case B2/B8	Case B5/B11
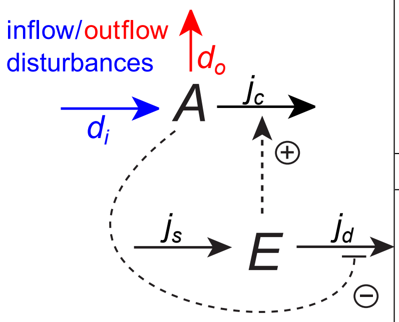	First order degradation of E	$j_c = k_c \cdot E \cdot A$<br>$j_s = k_s$<br>$j_d = k_d \cdot \frac{K_i^A}{K_i^A + A} \cdot E$	$j_c = k_c \cdot \frac{E}{K_a^E + E} \cdot A$<br>$j_s = k_s$<br>$j_d = k_d \cdot \frac{K_i^A}{K_i^A + A} \cdot E$
		Case B3/B9	Case B6/B12
	MM degradation of E	$j_c = k_c \cdot E \cdot A$<br>$j_s = k_s$<br>$j_d = k_d \cdot \frac{K_i^A}{K_i^A + A} \cdot \frac{E}{K_M^E + E}$	$j_c = k_c \cdot \frac{E}{K_a^E + E} \cdot A$<br>$j_s = k_s$<br>$j_d = k_d \cdot \frac{K_i^A}{K_i^A + A} \cdot \frac{E}{K_M^E + E}$

Motif 8		First order activation kinetics	Saturable activation kinetics
State equations		Case B1/B7	Case B4/B10
$\frac{dA(t)}{dt} = d_i(t) - d_o(t) - j_c(t)$<br>$\frac{dE(t)}{dt} = j_s(t) - j_d(t)$	Zero order degradation of E	$j_c = k_c \cdot \frac{K_i^E}{K_i^E + E} \cdot A$<br>$j_s = k_s \cdot \frac{K_i^A}{K_i^A + A}$<br>$j_d = k_d$	$j_c = k_c \cdot \frac{K_i^E}{K_i^E + E} \cdot A$<br>$j_s = k_s \cdot \frac{K_i^A}{K_i^A + A}$<br>$j_d = k_d$
Motif schematic		Case B2/B8	Case B5/B11
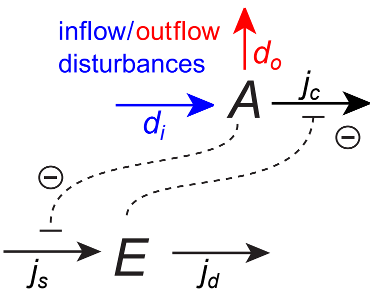	First order degradation of E	$j_c = k_c \cdot \frac{K_i^E}{K_i^E + E} \cdot A$<br>$j_s = k_s \cdot \frac{K_i^A}{K_i^A + A}$<br>$j_d = k_d \cdot E$	$j_c = k_c \cdot \frac{K_i^E}{K_i^E + E} \cdot A$<br>$j_s = k_s \cdot \frac{K_i^A}{K_i^A + A}$<br>$j_d = k_d \cdot E$
		Case B3/B9	Case B6/B12
	MM degradation of E	$j_c = k_c \cdot \frac{K_i^E}{K_i^E + E} \cdot A$<br>$j_s = k_s \cdot \frac{K_i^A}{K_i^A + A}$<br>$j_d = k_d \cdot \frac{E}{K_M^E + E}$	$j_c = k_c \cdot \frac{K_i^E}{K_i^E + E} \cdot A$<br>$j_s = k_s \cdot \frac{K_i^A}{K_i^A + A}$<br>$j_d = k_d \cdot \frac{E}{K_M^E + E}$

3 Antithetic controller motifs

In this section we show the complete set of system equations for the antithetic controller motifs shown in Fig 3 in the main paper. The equations are organized into tables for each motif where we include the general state equations, the schematics of each motif, and the detailed expressions for $j_c(t)$ and $j_{s,2}(t)$ for all cases. The inflow and outflow disturbances $d_i(t)$ and $d_o(t)$ (expressed as Eqs (3) and (4) in the main paper) along with $j_a(t)$ (shown in Eq (18) in the main paper) and $j_{s,1}(t){=}k_{s,1}$ are not included in the tables because they are unchanged between different motifs and cases. Disturbances marked in red in state equations and figure schematics are not included in the model for cases with one disturbance (cases A1–A2). Fig 4 from the main paper showing case descriptions is reposted here as Fig e for convenience.

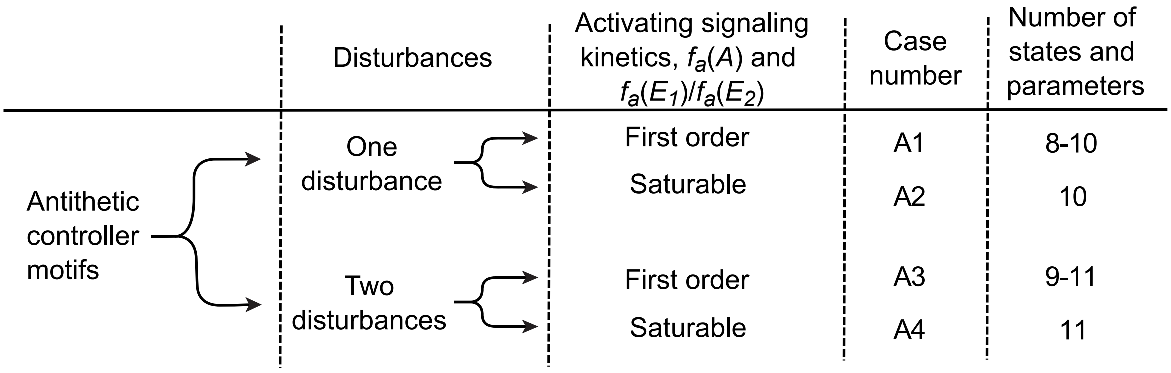

Fig e. Investigated cases of the antithetic motifs. Organization of the investigated cases for the antithetic controller motifs, taking into account the number of disturbances and the different expressions for the activation kinetics $f_a(A)$ and $f_a(E_1)/f_a(E_2)$. The combinations of these model complexities result in 4 cases named A1-A4, where the number of model parameters varies between 8 and 11.

<table>
<tr><th>Motif 1</th><th colspan="2">State equations</th></tr>
<tr><td rowspan="3">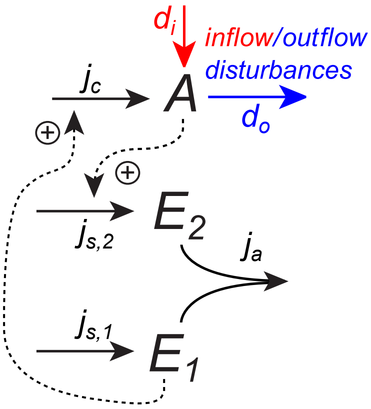
</td><td colspan="2">$$\frac{dA(t)}{dt} = d_i(t) - d_o(t) + j_c(t)$$ $$\frac{dE_1(t)}{dt} = j_{s,1}(t) - j_a(t)$$ $$\frac{dE_2(t)}{dt} = j_{s,2}(t) - j_a(t)$$</td></tr>
<tr><td>First order
activation kinetics
Case A1/A3</td><td>Saturable
activation kinetics
Case A2/A4</td></tr>
<tr><td>$$j_c = k_c \cdot E_1$$ $$j_{s,2} = k_{s,2} \cdot A$$</td><td>$$j_c = k_c \cdot \frac{E_1}{K_a^{E_1} + E_1}$$ $$j_{s,2} = k_{s,2} \cdot \frac{A}{K_a^A + A}$$</td></tr>
</table>

<table>
<tr><th>Motif 2</th><th colspan="2">State equations</th></tr>
<tr><td rowspan="3">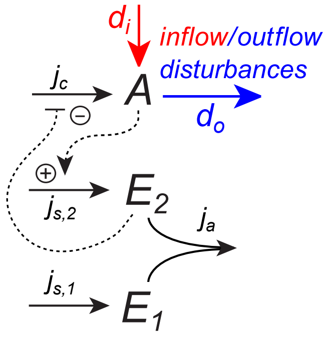
</td><td colspan="2">$$\frac{dA(t)}{dt} = d_i(t) - d_o(t) + j_c(t)$$ $$\frac{dE_1(t)}{dt} = j_{s,1}(t) - j_a(t)$$ $$\frac{dE_2(t)}{dt} = j_{s,2}(t) - j_a(t)$$</td></tr>
<tr><td>First order
activation kinetics
Case A1/A3</td><td>Saturable
activation kinetics
Case A2/A4</td></tr>
<tr><td>$$j_c = k_c \cdot \frac{K_i^{E_2}}{K_i^{E_2} + E_2}$$ $$j_{s,2} = k_{s,2} \cdot A$$</td><td>$$j_c = k_c \cdot \frac{K_i^{E_2}}{K_i^{E_2} + E_2}$$ $$j_{s,2} = k_{s,2} \cdot \frac{A}{K_a^A + A}$$</td></tr>
</table>

<table>
<tr><th>Motif 3</th><th colspan="2">State equations</th></tr>
<tr><td rowspan="3">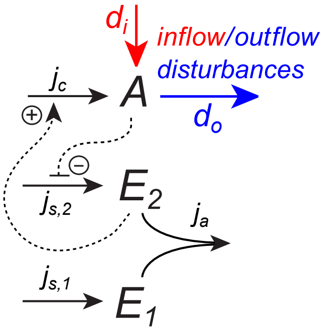
</td><td colspan="2">$$\frac{dA(t)}{dt} = d_i(t) - d_o(t) + j_c(t)$$
$$\frac{dE_1(t)}{dt} = j_{s,1}(t) - j_a(t)$$
$$\frac{dE_2(t)}{dt} = j_{s,2}(t) - j_a(t)$$</td></tr>
<tr><td>First order
activation kinetics
Case A1/A3</td><td>Saturable
activation kinetics
Case A2/A4</td></tr>
<tr><td>$$j_c = k_c \cdot E_2$$
$$j_{s,2} = k_{s,2} \cdot \frac{K_i^A}{K_i^A + A}$$</td><td>$$j_c = k_c \cdot \frac{E_2}{K_a^{E_2} + E_2}$$
$$j_{s,2} = k_{s,2} \cdot \frac{K_i^A}{K_i^A + A}$$</td></tr>
</table>

<table>
<tr><th>Motif 4</th><th colspan="2">State equations</th></tr>
<tr><td rowspan="3">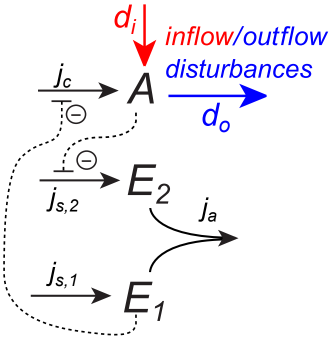
</td><td colspan="2">$$\frac{dA(t)}{dt} = d_i(t) - d_o(t) + j_c(t)$$
$$\frac{dE_1(t)}{dt} = j_{s,1}(t) - j_a(t)$$
$$\frac{dE_2(t)}{dt} = j_{s,2}(t) - j_a(t)$$</td></tr>
<tr><td>First order
activation kinetics
Case A1/A3</td><td>Saturable
activation kinetics
Case A2/A4</td></tr>
<tr><td>$$j_c = k_c \cdot \frac{K_i^{E_1}}{K_i^{E_1} + E_1}$$
$$j_{s,2} = k_{s,2} \cdot \frac{K_i^A}{K_i^A + A}$$</td><td>$$j_c = k_c \cdot \frac{K_i^{E_1}}{K_i^{E_1} + E_1}$$
$$j_{s,2} = k_{s,2} \cdot \frac{K_i^A}{K_i^A + A}$$</td></tr>
</table>

<table>
<tr><th>Motif 5</th><th colspan="2">State equations</th></tr>
<tr><td rowspan="3">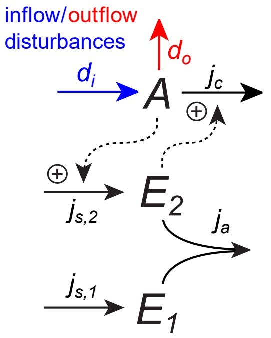
</td>
<td colspan="2">$$\frac{dA(t)}{dt} = d_i(t) - d_o(t) - j_c(t)$$ $$\frac{dE_1(t)}{dt} = j_{s,1}(t) - j_a(t)$$ $$\frac{dE_2(t)}{dt} = j_{s,2}(t) - j_a(t)$$</td></tr>
<tr><td>First order
activation kinetics
Case A1/A3</td><td>Saturable
activation kinetics
Case A2/A4</td></tr>
<tr><td>$$j_c = k_c \cdot E_2 \cdot A$$ $$j_{s,2} = k_{s,2} \cdot A$$</td><td>$$j_c = k_c \cdot \frac{E_2}{K_a^{E_2} + E_2} \cdot A$$ $$j_{s,2} = k_{s,2} \cdot \frac{A}{K_a^A + A}$$</td></tr>
</table>

<table>
<tr><th>Motif 6</th><th colspan="2">State equations</th></tr>
<tr><td rowspan="3">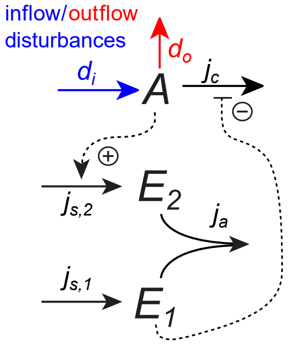
</td>
<td colspan="2">$$\frac{dA(t)}{dt} = d_i(t) - d_o(t) - j_c(t)$$ $$\frac{dE_1(t)}{dt} = j_{s,1}(t) - j_a(t)$$ $$\frac{dE_2(t)}{dt} = j_{s,2}(t) - j_a(t)$$</td></tr>
<tr><td>First order
activation kinetics
Case A1/A3</td><td>Saturable
activation kinetics
Case A2/A4</td></tr>
<tr><td>$$j_c = k_c \cdot \frac{K_i^{E_1}}{K_i^{E_1} + E_1} \cdot A$$ $$j_{s,2} = k_{s,2} \cdot A$$</td><td>$$j_c = k_c \cdot \frac{K_i^{E_1}}{K_i^{E_1} + E_1} \cdot A$$ $$j_{s,2} = k_{s,2} \cdot \frac{A}{K_a^A + A}$$</td></tr>
</table>

<table>
<tr><th>Motif 7</th><th colspan="2">State equations</th></tr>
<tr><td rowspan="3">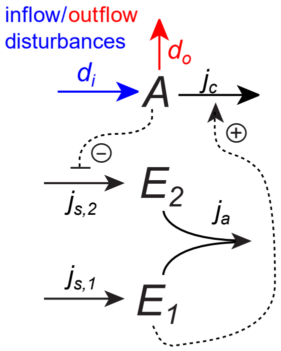
</td><td colspan="2">$$\frac{dA(t)}{dt} = d_i(t) - d_o(t) - j_c(t)$$ $$\frac{dE_1(t)}{dt} = j_{s,1}(t) - j_a(t)$$ $$\frac{dE_2(t)}{dt} = j_{s,2}(t) - j_a(t)$$</td></tr>
<tr><td>First order
activation kinetics
Case A1/A3</td><td>Saturable
activation kinetics
Case A2/A4</td></tr>
<tr><td>$$j_c = k_c \cdot E_1 \cdot A$$ $$j_{s,2} = k_{s,2} \cdot \frac{K_i^A}{K_i^A + A}$$</td><td>$$j_c = k_c \cdot \frac{E_1}{K_a^{E_1} + E_1} \cdot A$$ $$j_{s,2} = k_{s,2} \cdot \frac{K_i^A}{K_i^A + A}$$</td></tr>
</table>

<table>
<tr><th>Motif 8</th><th colspan="2">State equations</th></tr>
<tr><td rowspan="3">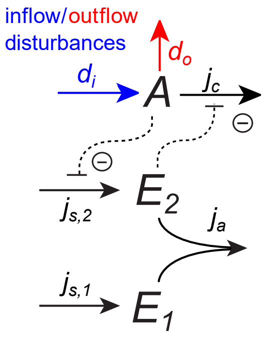
</td><td colspan="2">$$\frac{dA(t)}{dt} = d_i(t) - d_o(t) - j_c(t)$$ $$\frac{dE_1(t)}{dt} = j_{s,1}(t) - j_a(t)$$ $$\frac{dE_2(t)}{dt} = j_{s,2}(t) - j_a(t)$$</td></tr>
<tr><td>First order
activation kinetics
Case A1/A3</td><td>Saturable
activation kinetics
Case A2/A4</td></tr>
<tr><td>$$j_c = k_c \cdot \frac{K_i^{E_2}}{K_i^{E_2} + E_2} \cdot A$$ $$j_{s,2} = k_{s,2} \cdot \frac{K_i^A}{K_i^A + A}$$</td><td>$$j_c = k_c \cdot \frac{K_i^{E_2}}{K_i^{E_2} + E_2} \cdot A$$ $$j_{s,2} = k_{s,2} \cdot \frac{K_i^A}{K_i^A + A}$$</td></tr>
</table>
